# Perturbing plasma membrane lipid: a new paradigm for tumor nanotherapeutics

**DOI:** 10.7150/thno.82189

**Published:** 2023-04-23

**Authors:** Weidong Fei, Jingjing Yan, Xiaodong Wu, Shan Yang, Xiao Zhang, Rong Wang, Yue Chen, Junjun Xu, Caihong Zheng

**Affiliations:** 1 Department of Pharmacy, Women's Hospital, Zhejiang University School of Medicine, Hangzhou, 310006, China.; 2 Department of Gynecologic Oncology, Women's Hospital, Zhejiang University School of Medicine, Hangzhou, 310006, China.; 3 Department of Pharmacy, The Second Affiliated Hospital, Zhejiang University School of Medicine, Hangzhou, 310009, China.

**Keywords:** plasma membrane, nanotechnology, tumor, lipid, perturbing

## Abstract

Cancer is generally considered a result of genetic mutations that cause epigenetic changes, leading to anomalous cellular behavior. Since 1970s, an increasing understanding of the plasma membrane and specifically the lipid alterations in tumor cells have provided novel insights for cancer therapy. Moreover, the advances in nanotechnology offer a potential opportunity to target the tumor plasma membrane while minimizing side effects on normal cells. To further develop membrane lipid perturbing tumor therapy, the first section of this review demonstrates the association between plasma membrane physicochemical properties and tumor signaling, metastasis, and drug resistance. The second section highlights existing nanotherapeutic strategies for membrane disruption, including lipid peroxide accumulation, cholesterol regulation, membrane structure disruption, lipid raft immobilization, and energy-mediated plasma membrane perturbation. Finally, the third section evaluates the prospects and challenges of plasma membrane lipid perturbing therapy as a therapeutic strategy for cancers. The reviewed membrane lipid perturbing tumor therapy strategies are expected to bring about necessary changes in tumor therapy in the coming decades.

## Introduction

Despite significant advancements in cancer diagnosis and treatment in the past few decades, achieving favorable outcomes with current cancer therapies remains highly challenging. Therapeutic selectivity and resistance are the key recurrent issues observed during treatment 1. Novel treatment modalities for cancer have remained at the forefront of medical research in recent years.

Lipids, which are one of the primary components of cell membranes (also named plasma membrane), play an essential role in maintaining the normal lipid bilayer structure and biological membrane function. Lipids also play critical roles in organelle compartmentalization, cell signaling, protein storage, intercellular adhesion, and cell cycle regulation 2. While traditional cancer therapies mostly target proteins and nucleic acids in tumor cells, the potential use of lipid membranes in cancer diagnosis and treatment has been significantly overlooked, despite their well-known physicochemical characteristics and basic physiological functions that have been understood for several years. However, with a better understanding of the mechanisms involved in tumor occurrence and development, lipid interference therapy has emerged as a promising option for cancer treatment, given that lipids are involved in tumor progression 3.

The modulation of membrane lipid composition or interaction with lipid molecules can alter the lipidomic landscape, physical properties of membranes (e.g., fluidity and stiffness), and cellular signaling. Such changes in the structure and physicochemical properties of tumor cell membranes can significantly affect tumor development 4, 5. For example, targeting membrane fluidity can increase the susceptibility of ovarian cancer cells to auranofin (currently undergoing phase 2 clinical trials for epithelial ovarian cancer), which can stimulate DNA damage and enhance cellular oxidation 6. In addition, acetyl-CoA carboxylase (ACC) inhibitors, including soraphen A and ND646, have been shown to exhibit superior antitumor effects by significantly reducing fatty acid (FA) synthesis in tumor xenograft models 7. **Table 1** provides a summary of the membrane lipid perturbing drugs currently being tested in clinical trials for the treatment of cancer. Compared to other therapeutic strategies, membrane targeting strategies have many advantages. Firstly, the cell membrane is involved in various signal transduction pathways 8, and membrane disrupting molecules can interrupt multiple signaling pathways. Secondly, the cell membrane is tightly connected to the cytoskeleton to support the cell 9. Disrupting the cell membrane can lead to the leakage of cell contents, which cannot be repaired by metabolic compensation. Furthermore, changing the physicochemical properties of the membrane, such as altering the permeability of the membrane, can enhance the uptake of chemotherapeutic molecules 5.

The development of nanoscience has played an indispensable role in the field of tumor therapy 10. Currently, more than 50 types of nanodrugs have been approved for treating malignant tumors 11. Unlike many small molecule drugs that can enter cells through diffusion, nanodrugs have an inherent advantage in membrane lipid perturbation therapy, since they first interact with the cell membrane before being engulfed by cells. Over the past few decades, tumor membrane lipid nanotherapeutics have emerged due to the following reasons: 1) nanocarriers enable the targeted delivery of lipid-disrupting drugs (such as melittin), which prevents drug degradation and reduces side effects on non-target tissues 12-14; 2) many nanoparticles (NPs) exhibit prominent membrane cleavage effects. For example, nanomaterials with oxidative capacity (metal nanomaterials with Fenton catalysis ability), lipid-soluble nanodrugs, lipid-inserted materials, and nanomaterials with high cell membrane solubility can damage the structure of cell membranes and exert antitumor effects through oxidation, physical dissolution, and other deleterious effects (**Table 2**); 3) various nanostructures can affect the integrity of the cell membrane under the stimulation of external energy sources, such as light and ultrasound, and can even cause physical damage to the cell membrane, leading to the leakage of cellular contents (**Table 2**); 4) the ease of surface modification and the ability to adjust hardness allow nanoplatforms to selectively bind to different membrane domains. Rigid NPs tend to target the raft domain, while flexible NPs prefer the interface of the raft or non-raft domains 15. Future researchers could design nanodrugs with different rigidity to target different segments of tumor cell membranes.

The differential nature of cancer cell membranes has been reviewed in a few studies 5, 8, 16, but only a few studies have focused on the eminent role of nanotechnology in membrane lipid therapy 17. This review provides a comprehensive introduction, written in a concise form, which covers both the basic knowledge of attractive molecular targets and effective therapeutic strategies for future explorations. Compared with other related reviews, we established a connection between nanotechnology and molecular medicine in plasma membrane lipid perturbing for cancer therapy, providing perspectives on translational research using pharmacology approaches 16, 18, 19.

**Figure 1:**
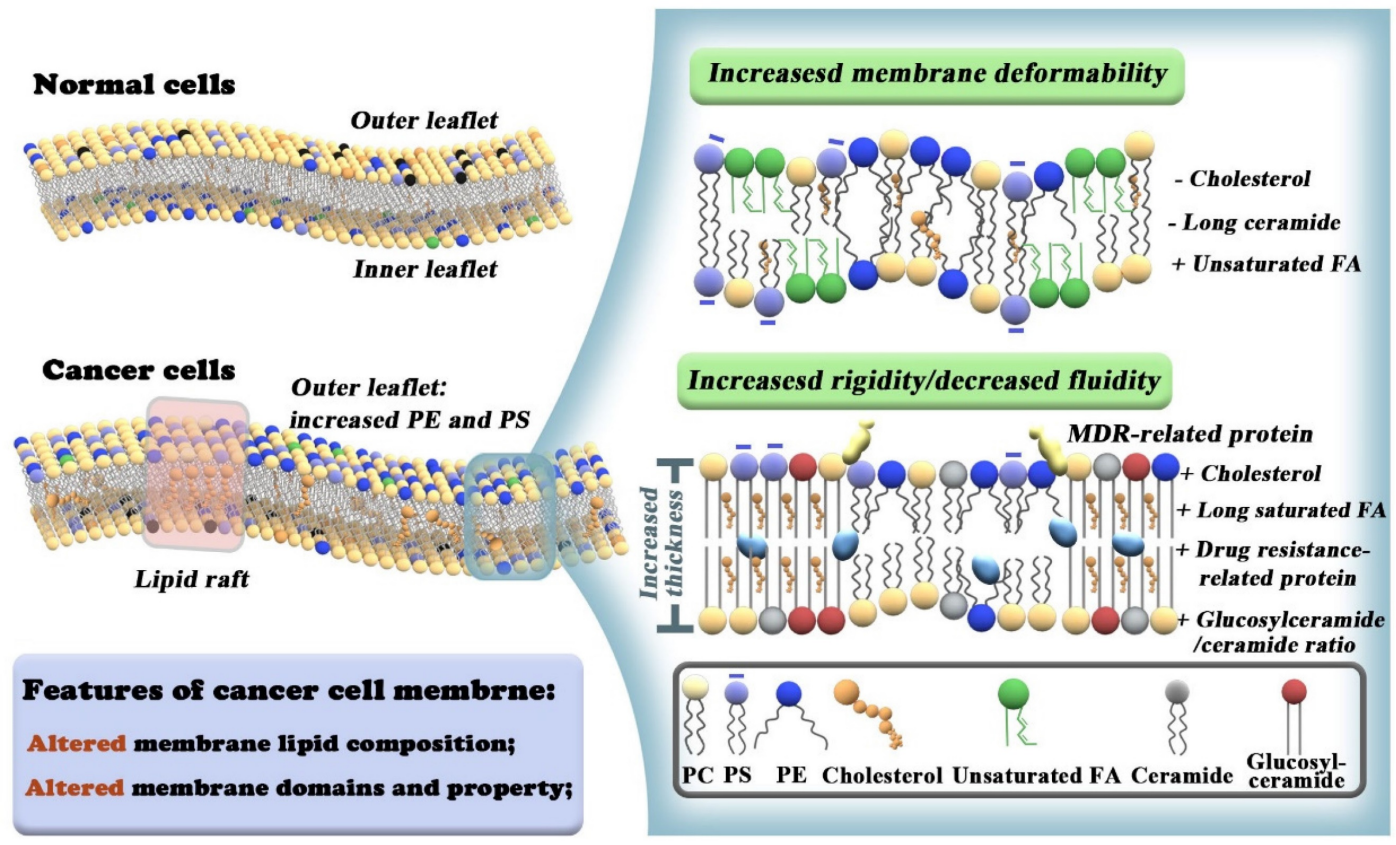
Differences between membrane lipid composition and organization in normal vs cancer cells. Cancer cells exhibit loss of trans bilayer asymmetry with increased PE and negatively charged PS lipids at the extracellular leaflet (left panel). Lower cholesterol and long-chain ceramide levels, higher polyunsaturated FAs exhibit membrane deformability and promote metastatic properties of cancer cells (upper right panel). Increased cholesterol levels and very long saturated FA chains contribute to high levels of ordered lipid raft domains and increased membrane rigidity of MDR cancer cells. A high glucosylceramide/ceramide ratio and overexpression of MDR-related proteins also lead to drug resistance (lower right panel).

**Figure 2:**
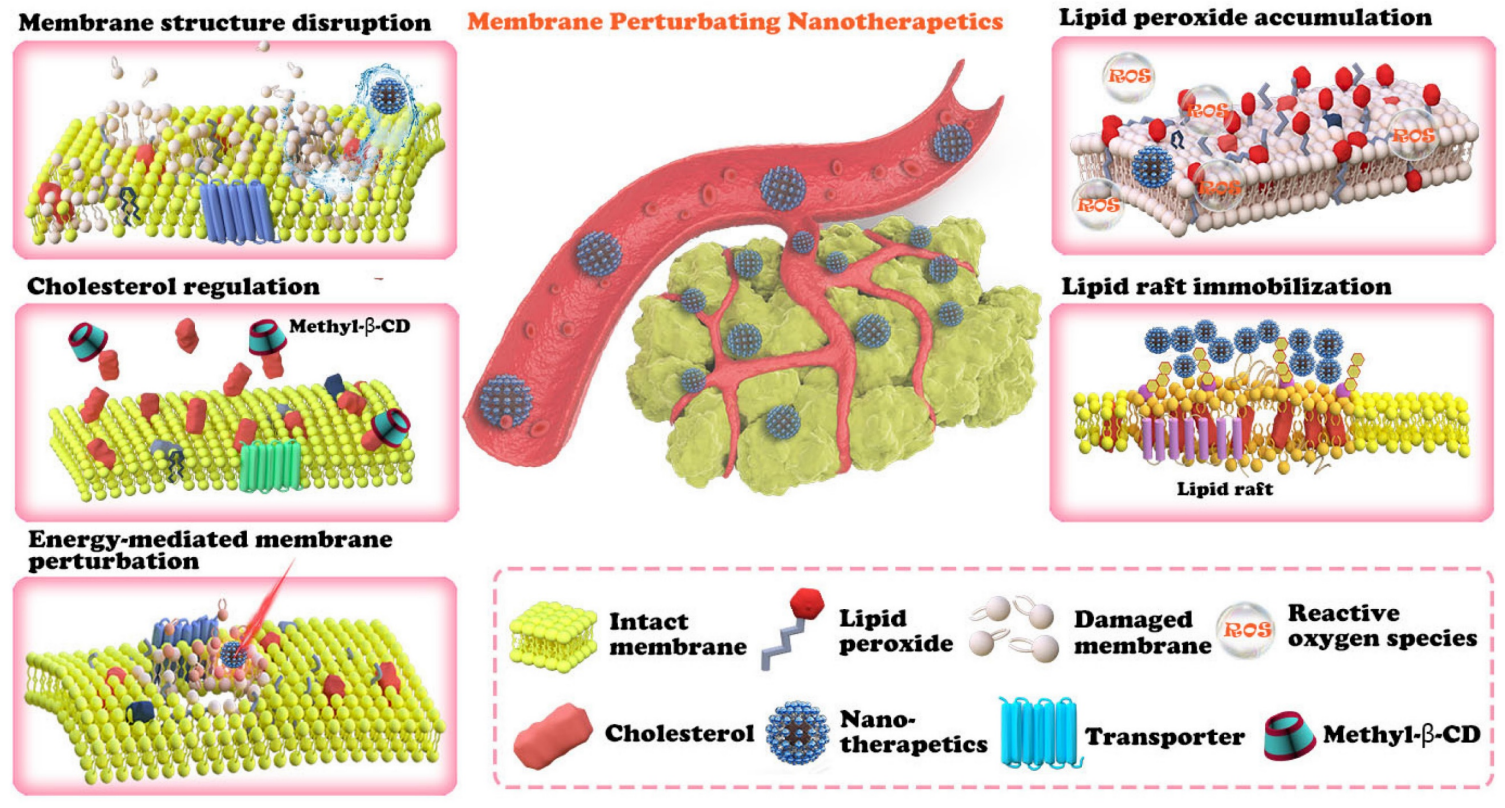
An overview of plasma membrane lipid perturbing tumor nanotherapeutics. The nanotechnology-involved plasma membrane lipid perturbing strategies described in this paper include lipid peroxide accumulation, cholesterol regulation, membrane structure disruption, lipid raft immobilization, and energy-mediated plasma membrane perturbation.

Furthermore, this paper contains a discussion of the future clinical application of such a therapeutic strategy, which is important but rarely discussed in other related reviews. The goal of this review is to promulgate the effectiveness of plasma membrane lipid perturbing tumor nanotherapeutics and attract more attention to this field of research. The first section of this paper introduces the characteristics of the tumor plasma membrane (**Figure 1**), including the two special lipid domains, and then demonstrates the link between plasma membrane composition and tumor signaling, metastasis, and drug resistance. This paper is then devoted to discussing the existing nanotherapeutic strategies used to perturb cell membranes and spotlighting the innate advantages of nanotechnology in membrane lipid therapy (**Figure 2 and Table 2**). We believe that this review could help researchers understand the multifaceted advantages of cell membrane disruption strategies and thus encourage the development of more efficient membrane lipid therapies.

## Characteristics of tumor plasma membranes

Advances in the new field of lipidomics and membrane biophysics have revealed the specificity of cancer cell membranes. They are not just simple barriers, but complex structures with amazing functions that even dictate the development and progression of cancer. The fundamental differences between cancer cell membranes and normal tissue cell membranes form the rationale for targeted membrane lipid perturbing therapy.

### Characteristics of tumor plasma membranes and their influence on tumor cells

For rapid adaptation to proliferation, survival, migration, or resistance to drug treatment, cancer cells need to reconstitute the plasma membrane through upregulated lipid metabolism genes and altered lipid metabolism. Lipid profiles on cancer cell membranes are cancer-specific and vary depending on the stage or susceptibility status of cancer. For example, breast cancer cell membranes overexpress cholesterol and its derivatives 20. Thus, cholesterol depletion drugs (such as statins) can be used to interfere with the function of the plasma membrane in breast tumors. Glioma can be treated by regulating the content of sphingomyelin and phosphatidylserines (PS) in the plasma membrane. Disturbance of lipid metabolism enzymes is a potential method for multiple myeloma therapy (as displayed in **Table 1**). In the future, membrane lipid perturbing therapy can be generalized to more tumors after analyzing the lipid profiles of different tumor cell membranes.

Lipids are the cornerstone of cell membranes, consisting of a polar head group and a relatively long hydrophobic tail. They can primarily be divided into three groups: glycerophospholipids, sphingolipids (SL), and sterols. The most common glycerophospholipids are PS, phosphatidylcholines (PC), phosphatidyl ethanolamines (PE), phosphatidyl inositols (PI), phosphatidyl glycerol (PG), and 1,2-diacylglycerol. Ceramides and glucosylceramides are important SL. Cancer cells show strong lipid avidity as evidenced by upregulated *de novo* fatty acids FA synthesis and enhanced extracellular lipid uptake, which is a prerequisite for membrane formation 8. The lipid bilayer is considered the basic structure of the plasma membrane. It is worth noting that the types and distributions of lipids in the membrane are heterogeneous. PS and PE are prevalent in the inner leaflet of the cell membrane under physiological conditions. In tumor cells, the trans-bilayer asymmetry is lost, with the enrichment of PS and PE on the outer leaflets. This provides an opportunity for targeted cancer cell therapy without affecting normal cells (**Figure 1**). In addition to lipid alterations, there is also overexpression of special protein receptors on the tumor cell membrane. The association of different membrane lipids and differential preferences between lipids and proteins contribute to diverse biological processes 3, such as signal transduction, molecular recognition, immune response, and cell proliferation and differentiation 21.

The plasma membrane comprises two main lipid domains: lipid rafts and caveolae 22. Lipid rafts are planar nano-scale structures (10-200 nm) 23, 24 enriched with SL and cholesterol in the outer leaflet 25. The binding of lipids to the plasma membrane is rapid, dynamic, and reversible, and it involves the interaction between the -OH groups of cholesterol and SL through hydrogen bonds and Van der Waals forces 26. As hyper-proliferating cells, cancer cells require high levels of cholesterol for membrane biogenesis and function 27. The increased presence of lipid rafts in tumor cells contributes to malignant transformation, uncontrolled growth, invasion/metastasis, and drug resistance 28, 29. Furthermore, the lipid rafts of tumor cell membrane allow the overexpression of growth factor receptors such as epidermal growth factor receptors, insulin-like receptors, and Sigma receptors 30, 31, as well as other factors or receptors associated with tumor progression and invasions such as integrin, adhesin, CD44, and CD24 28. An interesting study showed that when tyrosine kinase Src is located and activated within lipid rafts, cancer cells can escape from the protective mechanism of apical extrusion of normal cells and promote carcinogenesis 32.

Caveolae are highly hydrophobic and make up 20% of the plasma membrane. They contain a cystic cavity with a diameter of about 50-100 nm 33. Caveolae and lipid rafts are similar in lipid composition, and lipid rafts are always surrounded by caveolins in membrane structures with morphological discontinuity 34. Importantly, caveolin-1-mediated signal transduction affects numerous biological processes, including cell proliferation, invasion, and death during the cell cycle 8. A study reported that the 5-year survival rate of triple-negative patients with high levels of stromal caveolin-1 was significantly better than that of patients with moderate or absent levels of stromal caveolin-1 35.

Furthermore, the decrease of caveolin-1 in cancer-associated fibroblasts within gastric cancer patients predicted adverse clinical outcomes such as early recurrence and a low 5-year survival rate 36. These studies indicate that caveolin-1 is strongly associated with tumor development and prognosis.

In addition to the two specific lipid domains, the biophysical properties of the plasma membrane, including orderliness, fluidity, adhesion, and rigidity/elasticity, also play vital roles in controlling membrane diffusion 37, protein localization, lipid rafts interaction, and transmembrane protein signaling 38, 39. For example, advanced tumor cells are highly mobile due to decreased cholesterol and long-chain ceramides and increased levels of polyunsaturated fatty acids, increasing the likelihood of metastasis (**Figure 1**) 5.

Cancer cells have an unusual regulation of hydrogen ion dynamics driven by poor vascularity perfusion, regional hypoxia, and increased glycolysis. These forces synergize/orchestrate together to create extracellular acidity 40, 41. The cell membrane contains many types of negatively charged lipids (such as PS). An increase in extracellular acidity enables more hydrogen ions to neutralize the negative charge of phosphate heads in lipids. This masks their negative charge, causing the arrangement of lipids in the cell membrane to become more compact, which drives drug resistance in cancer as the drug cannot penetrate the cell membrane 41, 42. Moreover, drug-resistant tumor cells possess high levels of cholesterol and very long saturated fatty acid chains, which increases the hardness and rigidity of the plasma membrane, blocking drug entry into tumor cells 43. A high glucosylceramide/ceramide ratio may also contribute to drug resistance (**Figure 1**) 44.

The cytotoxic T lymphocytes (CTLs), the main subset of lymphocytes, develop from activated naive CD8^+^ T cells and exert cytotoxic effects on cancer cells. CTLs carry out their activity through the immune synapse (IS), which is formed as a polarized structure between CTLs and targeted cancer cells. The polarity of CTLs allows for the directional release of their killing machinery onto the target cells, leading to their apoptotic demise. Current knowledge has demonstrated that the assembly and function of IS is a complex event coordinated by the cytoskeleton and motor proteins 45. However, it is reasonable to speculate that the cancer plasma membrane may also play an important role in antitumor immunity by affecting the formation and function of IS. This is because local interactions between IS and cancer plasma membrane have been discussed 46, 47. This is an interesting topic for antitumor studies, which requires further and in-depth research.

### Membrane components affect transmembrane signal transduction

The plasma membrane, which serves as the scaffold for most cellular signaling transduction molecules, can regulate various pathways governing cell proliferation, survival, and migration 48. Alterations in specific membrane lipid metabolism significantly impact signaling events in carcinogenesis. Lysophosphatidic acid (LPA), a low molecular weight bioactive phospholipid, acts as a potent modulator in tumor progression via binding to the endothelial differentiation gene family of G protein-coupled receptors (referred to as LPA1-3) on the plasma membrane 49. *In vitro* experiments have shown that LPA promotes the proliferation of endometrial carcinoma HEC-1A cells at physiological concentrations (0.1 and 1.0 μM), which may be attributed to the specific activation of LPA2 receptor or the presence of the switch of reciprocal receptor activity of different regulators 49. LPA2 receptors are also aberrantly upregulated in ovarian cancer cells compared to normal cells. The activation of AMP-activated protein kinase triggered by LPA via the LPA_2_ receptor contributes to dynamic cytoskeleton rearrangements and increased tumor metastasis 50.

Lipid rafts, abundant in cholesterol, are necessary for transforming growth factor (TGF)-β-mediated-epithelial-mesenchymal transition (EMT) 51. Jin *et al.* found that simvastatin, a cholesterol-lowering drug, can damage cholesterol-dependent lipid rafts microdomains, suppress the integrin β3/FAK pathway, and subsequent local adhesion formation. In addition, simvastatin can repolarize tumor-associated macrophages (TAMs) and promote the M2-M1 phenotypic transformation via cholesterol-related liver X receptors/adenosine triphosphate (ATP)-binding cassette (ABC) transporter A1 regulation, which upregulates tumor necrosis factor-α and downregulates TGF-β, thus remodeling the tumor microenvironment (TME) and synergistically reversing EMT-associated paclitaxel (PTX) resistance 52. Another study revealed that cholesterol deficiency attenuated ATK signal transmission and re-sensitized ductal adenocarcinoma to gemcitabine 48. In addition to drug resistance, the cholesterol present in lipid rafts also regulates metastasis. Yang *et al.* reported that squalene synthase, a cholesterol biosynthesis enzyme, stimulates the activation of Src and ERK1/2 through secreted phosphoprotein 1, resulting in increased expression of matrix metalloproteinase 1 and subsequent lung tumor metastasis 53. Conversely, lipid domains rich in long-chain ceramide are embedded with Fas/CD95 death receptors to promote apoptosis 5. As mentioned above, growth factor receptors and other functional proteins residing in lipid rafts participate in corresponding signal transduction to regulate the proliferation, differentiation, metastasis, or apoptosis of tumor cells.

### Membrane phospholipid composition affects metastasis of tumor cells

The most fundamental property of the plasma membrane is its fluidity, which controls the dynamics of the membrane domain and regulates lipid-protein separation and downstream cellular signaling processes 54. These processes include polarization, transport, cell growth, migration, and pathogen invasion 55. Accumulating evidence confirms that alteration in membrane fluidity has the potential to intervene in tumor malignancy, metastasis, or drug resistance 8. For instance, the antimetastatic ACC inhibitor soraphen A can interfere with FA synthesis and reduce highly aggressive cancer cell migratory capacity by increasing membrane rigidity 56. Additionally, diclofenac was confirmed to be effective in the chemoprevention of early colon cancer by reversing the increased membrane fluidity and dynamics induced by 1,2-Dimethyl-hydrazine dihydrochloride 57. Further, the lipophilic celecoxib also halted the metastatic potential of colon cancer cells by reducing plasma membrane fluidity 58. Based on the above studies, researchers can change the fluidity of tumor cell membranes by regulating the synthesis of FAs or introducing certain drugs with considerable lipid affinity.

Plasma membrane fluidity primarily depends on the levels of cholesterol, phospholipid species, and the content of unsaturated fatty acids. Cholesterol-deficient cells are more deformable plasma membranes that can freely enter the vasculature, thus conferring them with a higher metastatic potential 29. Saturated lipids with their straight hydrophobic tails are conducive to the ordered piling up of membranes. In contrast, unsaturated lipids occupy a slightly wedge-shaped space and disturb the dense packing of lipids, thereby increasing fluidity 59-61. A lipidomic study using a urothelial cell model compared the differences between non-metastatic (RT4) and metastatic (T24) plasma membranes. The study revealed that T24 cells have greater length and increased levels of ω-6 polyunsaturated FAs acyl chains, together with decreased levels of shorter, saturated, or mono-unsaturated lipid species 62. It is worth emphasizing that the membrane fluidity of tumor cells does not always have a positive correlation with their metastatic capacity. In most cases, the forces that drive cell migration are generated by cytoskeletal actin coupling 63. A definite rigidity of the cell membrane is required to maintain different cell shapes during this process. Therefore, our group hypothesized that tumor cells express appropriate levels of polyunsaturated fatty acids, which increase membrane fluidity, and cholesterol, which decreases membrane fluidity to allow the plasma membrane to exhibit the most suitable fluidity for metastasis. Researchers used high-performance liquid chromatography, to determine the qualitative and quantitative lipid composition in certain tumor membranes. For example, in colon cancer, the malignant transformation was accompanied by an increase in the amount of arachidonic and oleic acids related to lipid peroxidation 64. However, few studies currently describe the changing processes of membrane compositions during the different developmental stages of tumors. Furthermore, the composition of membranes strongly depends on the cancer type, with high inter-tumor variability. To design more powerful antitumor strategies, future research could investigate the changes in polyunsaturated fatty acids and cholesterol during the developmental processes of different tumors.

The adhesion of tumor cells to vascular endothelium and the basement membrane matrix is a key step in tumor invasion and metastasis. The membrane phospholipid composition of tumor cells is closely related to cell adhesion. The PS and PI within the plasma membrane contribute to surface charge. PS and PI constitute 15% and 10% of the lipids within the leaflets of the plasma membrane, respectively 65. They are isolated into many tiny regions by polycationic proteins that increase their local accumulation. The anionic phospholipids accumulated on the plasma membrane can form a reticular electric field with an intensity of about 10^5^ V/cm, attracting cationic substances such as proteins and ions 66. Tumorigenesis can cause an elevation of PS and PI levels, thereby affecting surface potential 67. These alterations could influence the localization of surface-charge-sensitive signaling proteins 65. For instance, the spatiotemporal regulation of surface charge may have a significant impact on the signaling molecules K-ras which is important in cancer biology. Failure of signaling cascade localization could be an important contributor to the progression from a normal cell to a neoplasm 68. Compared to normal cells, many tumor cell lines display a higher number of PS on the outer side of their membranes. The level of PS exposure is positively correlated with the degree of malignancy and the metastatic ability of malignant melanoma 3. Interestingly, some drug-free nanocarriers with PS targeting ability, such as zinc(II)- diphenylamine-modified liposomes or phosphatidylcholine-stearyl amine, exhibit potent anticancer activity 69, 70. Saponin C-modified liposomes have presented compelling clinical results and an impressive safety profile in Phase I clinical trials 70. These therapeutic agents that target specific lipid components of cancer cell membranes indicate their value as an important addition to cancer therapy.

### The relationship between membrane phospholipid composition and drug resistance in tumors

The increase in cholesterol levels in the plasma membrane is closely linked to drug resistance. Multi-drug resistant (MDR) cells tend to exhibit a more rigid plasma membrane, which results in decreased cell permeability 29, 71. Compared to drug-sensitive cells, MDR cells show higher levels of cholesterol and special types of phospholipids (PC, PI, PE, etc.), and a 60% increase in protein/lipid ratio 17, 29, 72. The low permeability of MDR cells limits drug entry into tumor cells, which partially explains their resistance to therapy 29, 73, 74. A recent study reduced the cholesterol content in the plasma membrane with statins, thereby re-sensitizing tumor cells to doxorubicin (DOX) 75.

Other studies have shown that the phenotypes of drug resistance in tumors are associated with lipid upregulation in lipid rafts and caveolae 76. On the one hand, the abundance of lipid raft domains has been found to correlate with the invasive potential and membrane fluidity of tumor cells 71. On the other hand, the lipid rafts of tumor cells contain several MDR transporters, including P-glycoprotein (P-gp) and other MDR-related proteins that pump out chemotherapeutic drugs from the cytoplasm. Thus, the lipid raft domains play a vital role in the development of MDR in tumor cells. Furthermore, recent evidence highlights the important role of caveolin-1 and P-gp in intracellular cholesterol transport to the plasma membrane 77. As an epigenetic drug inhibiting DNA methylation, 5-aza-2'-deoxycytidine can deplete cholesterol-sphingomyelin rafts, alter lipid profiles, and reverse drug resistance 78.

Particularly, the expression of caveolin-1 on the plasma membrane strongly impacts chemotherapeutic resistance 79. It has been shown that caveolin-1 levels are significantly downregulated in A549 lung cancer cells after cisplatin exposure. Furthermore, the knockdown of cavelin-1 promotes cisplatin-induced cell death in A549 cells 79. Mechanistically, the inhibition of caveolin-1 induces the release of mitochondrial reactive oxygen species (ROS), the destruction of cell metabolism, and the decrease in mitochondrial membrane potential, thus amplifying the mitochondrial stress signals induced by cisplatin. Caveolin-1 overexpression has also been documented in 5-fluorouracil-resistant colon cancer cells 80, cisplatin-resistant ovarian cancer cells 81, PTX-resistant breast and lung cancer cells 82, as well as in trastuzumab emtansine-resistant gastric cancer cells and human epidermal growth factor receptor 2-positive breast cancer cells 83. Hence, the therapeutic efficacy of small molecular chemotherapeutic drugs could be improved by decreasing caveolin-1 expression. For example, the inhibition of caveolin-1 by β-cyclodextrin can re-sensitize resistant colon cancer cells to 5-fluorouracil 80.

In general, MDR tumor cells show an altered composition of membrane phospholipids and glycosphingolipids and overexpress long saturated fatty acid chains and cholesterol. Such characteristics reduce membrane fluidity and alter the spatial structure and interaction with the transmembrane helices of the ABC transporters, thus facilitating drug binding and drug efflux 44. Moreover, the higher proportion of monosaturated/polyunsaturated fatty acids in drug-resistant tumor cells reduces the production of reactive aldehydes with cytotoxic and pro-inflammatory activities, limiting lipid peroxidation-induced damage in tumors 44.

The identification of new targets for antitumor drugs remains a pressing need. Lipids regulate the normal physiological activities of the plasma membrane and participate in signal transduction. Investigating the relationship between the membrane and tumorigenesis is becoming a new area of interest in cancer therapy. The discussion above highlights many antitumor targets and strategies based on the perturbation of the plasma membrane, which is also known as “membrane lipid therapy”. Besides regulating the content of special lipids (such as LPA, PS, and PI) and cholesterol, disrupting the synthesis, metabolism, localization, and transport of ordinary lipids can also achieve therapeutic goals 8. Although the specific composition and function of tumor plasma membranes have received little attention in the past, tumor treatment strategies based on plasma membrane disruption will gradually gain interest in cancer treatment as our understanding of tumor plasma membranes and the characteristics of related lipids deepens.

## Plasma membrane lipid perturbing tumor nanotherapeutics

As mentioned earlier, the alteration in the lipid composition of tumor cells and their effects on cell function have made membrane lipid therapy an exciting prospect for the specific targeting of cancers. In this section, we will present nanotechnology-based plasma membrane lipid perturbation strategies.

### Plasma membrane lipid peroxide accumulation tumor nanotherapeutics

**Figure 3:**
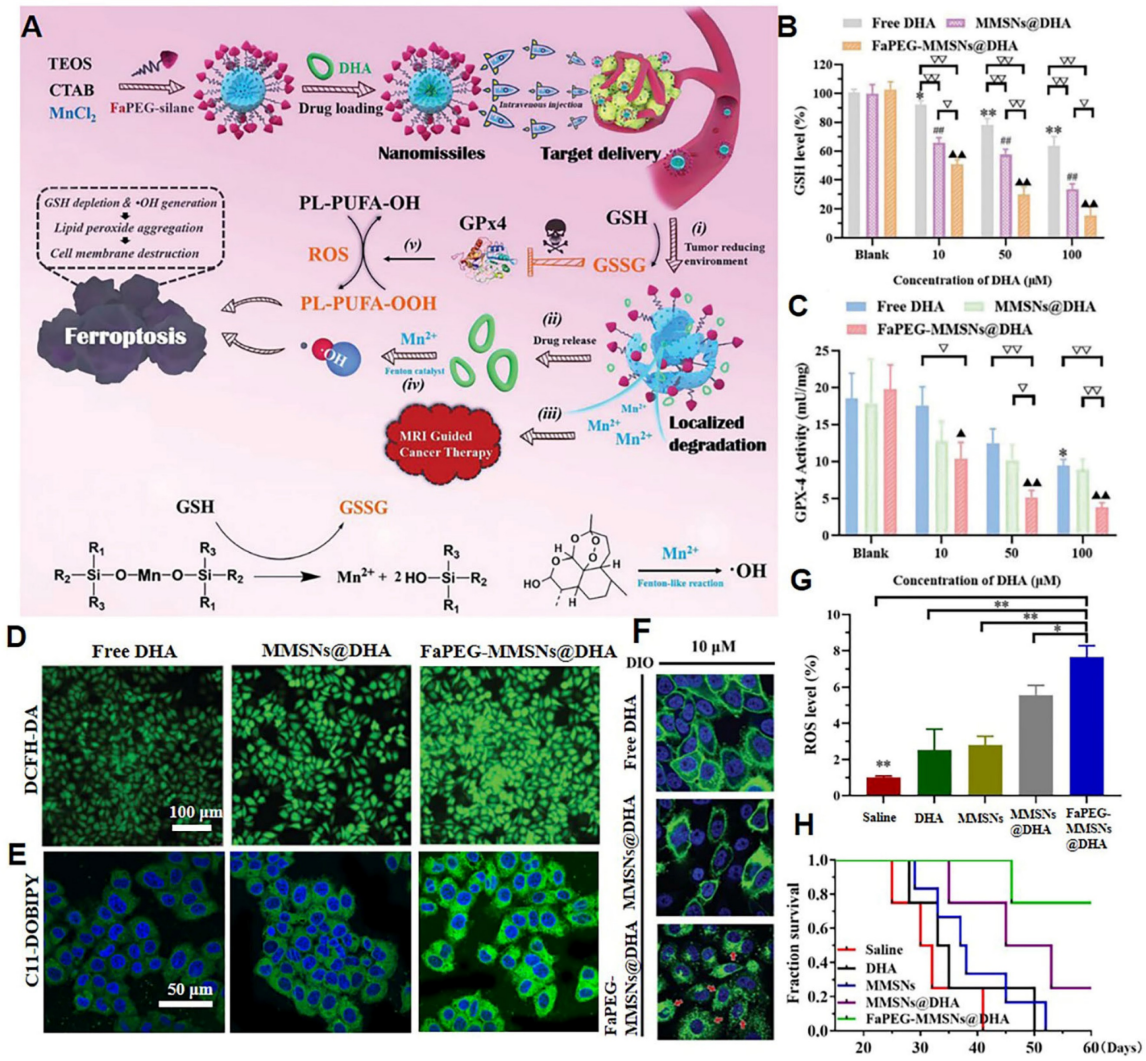
Nanomissile-mediated lipid peroxide accumulation in tumor cell membrane. (A) Schematic illustration of the construction of nanomissiles and the ferroptosis-inducing mechanism by GSH exhaustion and ·OH self-production. (B) GSH levels and (C) GPx4 activity of HepG2 cells after indicated treatment. (D) ROS and (E) lipid peroxide detection of HepG2 cells stained with DCFH-DA, C11-BODIPY^581/591^ after co-incubation with different formulations at same DHA concentration. (F) DIO-labeled cell membrane morphology changes after co-incubation with different formulations at same drug concentration. (G) Enhanced ROS generation in the tumor site. (H) The survival curve of mice after various treatments. (Adapted with permission from Ref. 87. Copyright 2020 Royal Society of Chemistry)

Lipid peroxidation products significantly affect the structure and dynamic properties of the cell membrane. The increase in lipid peroxides disturbs the order of lipids within the phospholipid bilayer, leading to the formation of pores. The entry of reactive substances, such as ROS and reactive nitrogen species within tumor cells leads to oxidative damage to intracellular macromolecules, such as DNA and proteins 84. Glutathione peroxidase 4 (GPx4) is an enzyme that is highly expressed in tumor cells and is capable of reducing lipid peroxides to unsaturated fatty acids, thereby antagonizing the damage caused by lipid peroxides accumulation in the tumor plasma membrane 85. The activation of GPx4 depends on intracellular glutathione (GSH). In 2019, our group designed a sorafenib-loaded nanodrug that specifically released sorafenib to inhibit the synthesis of GSH and upregulate the content of lipid peroxide in tumor cells 86. Such nanoplatforms can significantly inhibit the proliferation of tumor cells. In 2020, our group achieved the rapid aggregation of lipid peroxides in tumor cell membranes by deactivating GPx4 and enhancing ROS generation in tumor cells 87. To accomplish this, we constructed folate-polyethylene glycol (PEG)-modified manganese-doped mesoporous silica NPs loaded with dihydroartemisinin (DHA) (referred to as nanomissiles) for MRI-guided ferroptosis tumor therapy (**Figure 3A**). Intracellular GSH triggers the degradation of nanomissiles, leading to intracellular GSH depletion and strong inhibition of GSH-dependent GPx4 activity **(Figure 3B-C)**. Subsequently, the simultaneous release of DHA and Fenton catalysis Mn^2+^ contributes to hydroxyl radicals (·OH) production (**Figure 3D**). These multi-angle effects accelerate the oxidation of polyunsaturated fatty acids to toxic lipid peroxides (**Figure 3E**). Due to the deleterious effect of accumulated lipid peroxides, the cell membrane dissolved and disappeared **(Figure 3F)**. In the *in vivo* study, nanomissile-treated mice achieved the highest ROS level in tumor tissue (**Figure 3G**) and the longest survival time (**Figure 3H**). Our research also included a hemolysis study, H&E-stained images of major organs, serum biochemical parameters, and body weight curves, all of which demonstrated the biosafety of the designed nanoplatforms. In general, our study demonstrated the potential to disrupt cell membrane integrity via lipid peroxide accumulation to achieve anticancer effects.

### Plasma membrane cholesterol regulation tumor nanotherapeutics

#### Cholesterol clearance tumor nanotherapeutics

**Figure 4:**
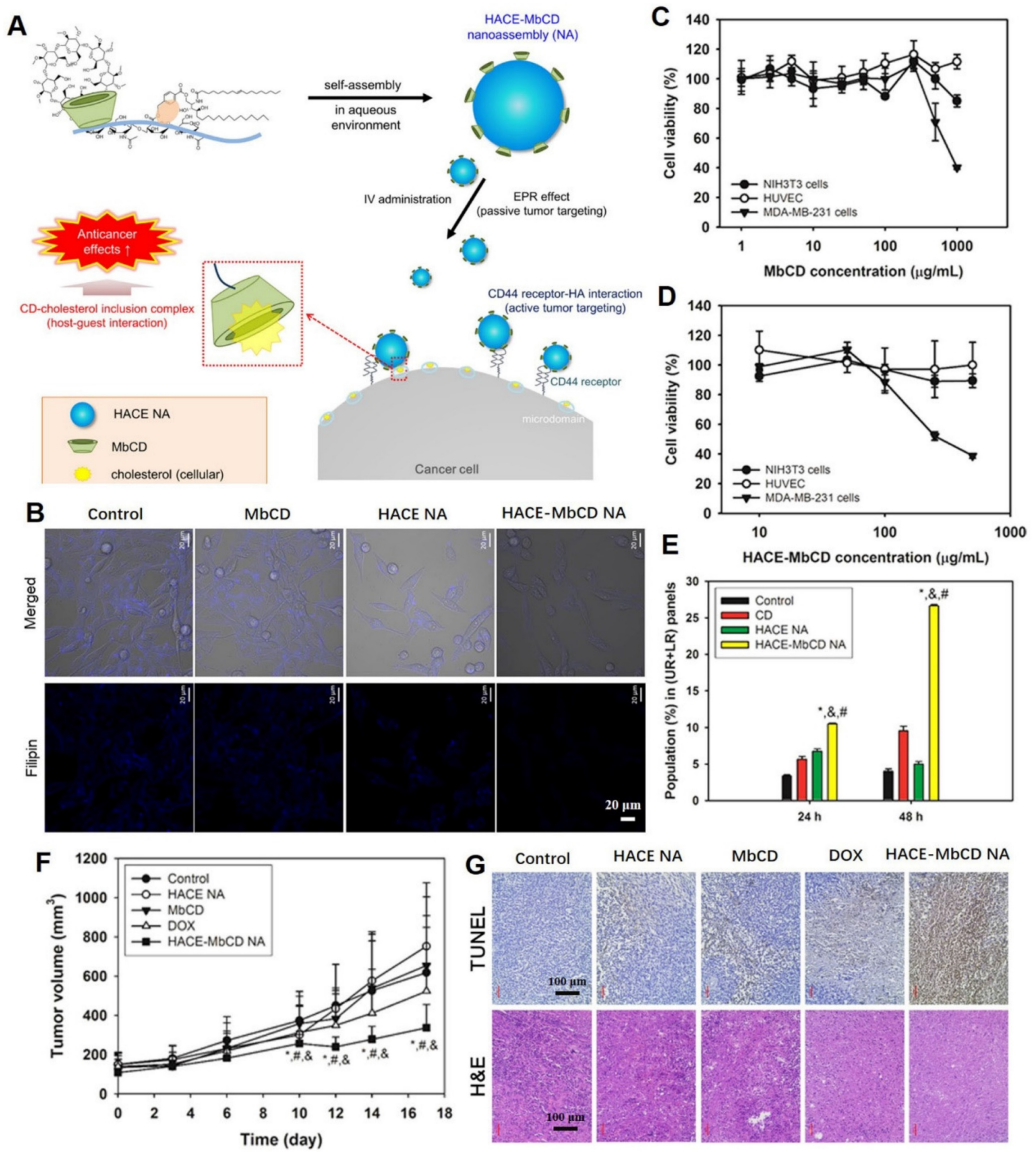
Tumor-targeted nanoassemblies for cholesterol extraction by MbCD. (A) Schematic illustration regarding tumor targeting and cellular cholesterol-capturing strategy of HACE-MbCD NA. (B) Cholesterol capture capacity, detected by filipin III-stained free cholesterol (blue color), of HACE-MbCD NA in MDA-MB-231 cells. Antiproliferation assay of (C) MbCD and (D) HACE-MbCD in NIH3T3, HUVEC, and MDA-MB-231 cells. (E) Apoptotic efficacies of HACE-MbCD NA in MDA-MB-231 cells. Population percentages of upper right (UR) and lower right (LR) quadrants are presented. (F) MDA-MB-231 tumor volume profiles of different groups. (G) Microscopic images of dissected tumors after H&E and TUNEL stainings. (Adapted with permission from Ref. 90. Copyright 2018 American Chemical Society)

Cholesterol is the key molecule in lipid rafts, and its removal can rupture lipid rafts and caveolae, resulting in protein shedding, defuctionalization and deregulated cell signaling 88, 89. Nanoplatforms can significantly improve drug delivery, increase tumoral selectivity, reduce adverse effects and target plasma membrane components in tumor therapy. In a previous study, the cholesterol-capturing moiety, methyl-β-cyclodextrin (MbCD), was covalently bonded to an amphiphilic hyaluronic acid-ceramide (HACE) structure, and self-assembled into a spherical nanoassembly (named HACE-MbCD NA) of 198 nm size with a unimodal distribution **(Figure 4A)** 90. Due to CD44 receptor-HA interaction-mediated active tumor targeting effects, the HACE-MbCD NA efficiently extracted cholesterol from tumor cell membranes when compared to MbCD **(Figure 4B).** After treatment with HACE-MbCD NA, the MDA-MB-231 cells exhibited greater apoptosis and low proliferation rates when compared to noncancer cells **(Figure 4C-E)**. This result demonstrated that the prepared nanoplatforms were less toxic to noncancer cells. The anticancer effects of HACE-MbCD were comparable to those of DOX, but showed less individual variations and were more consistent across tumor-xenografted mice models **(Figure 4F)**. The HA-CD44 binding affinity and higher cholesterol extraction capacity of HACE-MbCD promoted the apoptosis events **(Figure 4G)**. The H&E-stained slice images of major organs and serum biochemical parameters supported the biosafety of the designed nanoplatforms in cancer therapy.

#### Cholesterol metabolism regulation tumor nanotherapeutics

Drug delivery systems are specifically tailored to intervene cholesterol metabolism. A TME-activatable penetration peptide-modified multifunctional liposome significantly enhanced the therapeutic efficacy of simvastatin by regulating cholesterol metabolism to reverse EMT and TAM repolarization and successfully reversed EMT-related drug resistance 52. Alternatively, both DOX and simvastatin were encapsulated in α-tocopherol polyethylene glycol 2000 succinate-modified poly (lactic-glycolic acid) (PLGA) NPs. The drug delivery system achieved synergistic simvastatin-mediated cholesterol consumption and P-gp downregulation in lipid rafts, which disrupted the packing density of lipid rafts and suppressed drug efflux. The NPs also promoted mitochondrial apoptosis and re-sensitized DOX-resistant SW620/AD300 cells. Such therapeutic strategy could serve as a prospective candidate for reversing drug resistance via simvastatin-loaded nanoplatforms and its cholesterol depletion capacity in lipid rafts 91.

Another nanosystem, consisting of chondroitin sulfate shell and metal organic framework (NH_2_-MIL-88B) core, consumes cholesterol through cascade catalysis. The NH_2_-MIL-88B — rich in amino groups — successfully immobilized cholesterol oxidase via an amide reaction, increasing enzyme tolerance to high temperatures and low pH. Cholesterol oxidase catalyzes cholesterol oxidation, and its metabolite (H_2_O_2_) is further catalyzed by metal organic frameworks with peroxidase activities to generate ·OH. Effective cholesterol level reduction in drug-resistant cell membranes promoted DOX cellular uptake. The generation of ·OH after cholesterol oxidation was favorable for tumor inhibition, turning “trash” into “treasure” for maximum treatment efficiency. The chondroitin sulfate shell specifically targeted tumor cells via CD44 receptors and suppressed intracellular anti-apoptotic protein (Bcl-XL) levels, increasing tumor cell sensitivity to chemotherapeutics 92.

#### Other cholesterol regulation tumor nanotherapeutics

Cholesterol is essential for forming T cell receptors on cytotoxic CD8^+^ T lymphocytes and activation of CD8^+^ T cells 93. Activated T cells upregulate acyl-CoA cholesterol acyltransferase-1 (ACAT-1) levels, leading to cholesterol esterification and attenuation of immune effects 94. Thus, antitumor activities can be enhanced by a combination of ACAT-1 inhibition with chemotherapy or immunotherapy. Co-encapsulation of PTX and the immunoadjuvant (αGC) in liposomes was performed. Then, the liposomes were modified with a pH-sensitive TH peptide (PTX/αGC-TH-Lip) to realize chemo-immunotherapeutic effects. Combined with avasimibe (an ACAT-1 inhibitor), PTX/αGC-TH-Lip enhanced CTL responses with elevated levels of free cholesterol and ameliorated the inhibition of CD8^+^ T cells in melanoma xenografts and lung metastasis models. Cholesterol-associated metabolic regulation also acted as a key link in mediating antitumor immunity 94.

Cholesterol uptake in malignant lymphoma cells is dependent on lipoprotein-mediated transport. Selective blockade of cholesterol uptake by cholesterol-addicted cells results in lipid peroxides accumulation, increasing their vulnerability to ferroptosis. Researchers treated lymphoma cell lines with cholesterol-poor high-density lipoprotein-like NPs to activate compensatory metabolic responses and *de novo* cholesterol synthesis, accompanied by suppressed GPx4 expressions that contribute to cell death via a mechanism consistent with ferroptosis 95. Elevated serum cholesterol levels are positively associated with increased cancer risk in melanoma, prostate cancer, non-Hodgkin's lymphoma, endometrial and breast cancers 96. The association between cholesterol uptake and ferroptosis provides a promising therapeutic target for these specific cancers 95. Since cholesterol is important in lipid rafts, the nanoplatform focusing on cholesterol depletion or metabolism regulation will exert targeted anticancer effects and reverse drug resistance in cancers with high cholesterol levels, such as breast, non-small cell lung and prostate cancers. In summary, membrane cholesterol regulation-based therapies are among the most commonly used and effective anticancer strategies.

### Membrane structure disruption tumor nanotherapeutics

Membranolytic molecules induce detergent-like plasma membrane rupture and result in release of cellular contents by interacting with phospholipids, which constitute the main cell membrane skeleton. Membrane rupture strategies are effective against MDR cells, however, given the importance of preventing membranolytic effects in normal cells, low tumor selectivity is a major challenge. Liu *et al*. designed a library of “proton transistors” consisting of a PEG block group and a pH-responsive membranolytic block group, which could switch slight pH perturbation signals to drastic changes for membranolytic activities, enabling selective membrane rupture within the slightly acidic TME 97. The best nanodetergent was P(C6-Bn_20_), which enhanced cytotoxicity by more than 32-fold when the pH changed by 0.1 (“on state”), while self-assembling into neutral NPs at physiological pH (“off state”). At pH 6.8, the membranolytic block of P(C6-Bn_20_) was protonated and bound the negatively charged PS on lipid membranes. The cation-π interactions facilitated benzyl group insertion into the membrane generated many holes, resulting in rapid release of ATP and lactate dehydrogenase. At pH 6.8, ferrocene-labelled P(C6-Bn_20_) co-localized with the cell membrane, and cell morphology disruptions were observed, with blebbing. *In vivo* antitumor assays revealed that P(C6-Bn_20_) dose-dependently suppressed Panc02 tumor growth. A much paler H&E staining implied the leakage of tumor cell contents. The H&E-stained slice images of major organs exhibited low toxicity of P(C6-Bn_20_) on normal tissues.

**Figure 5:**
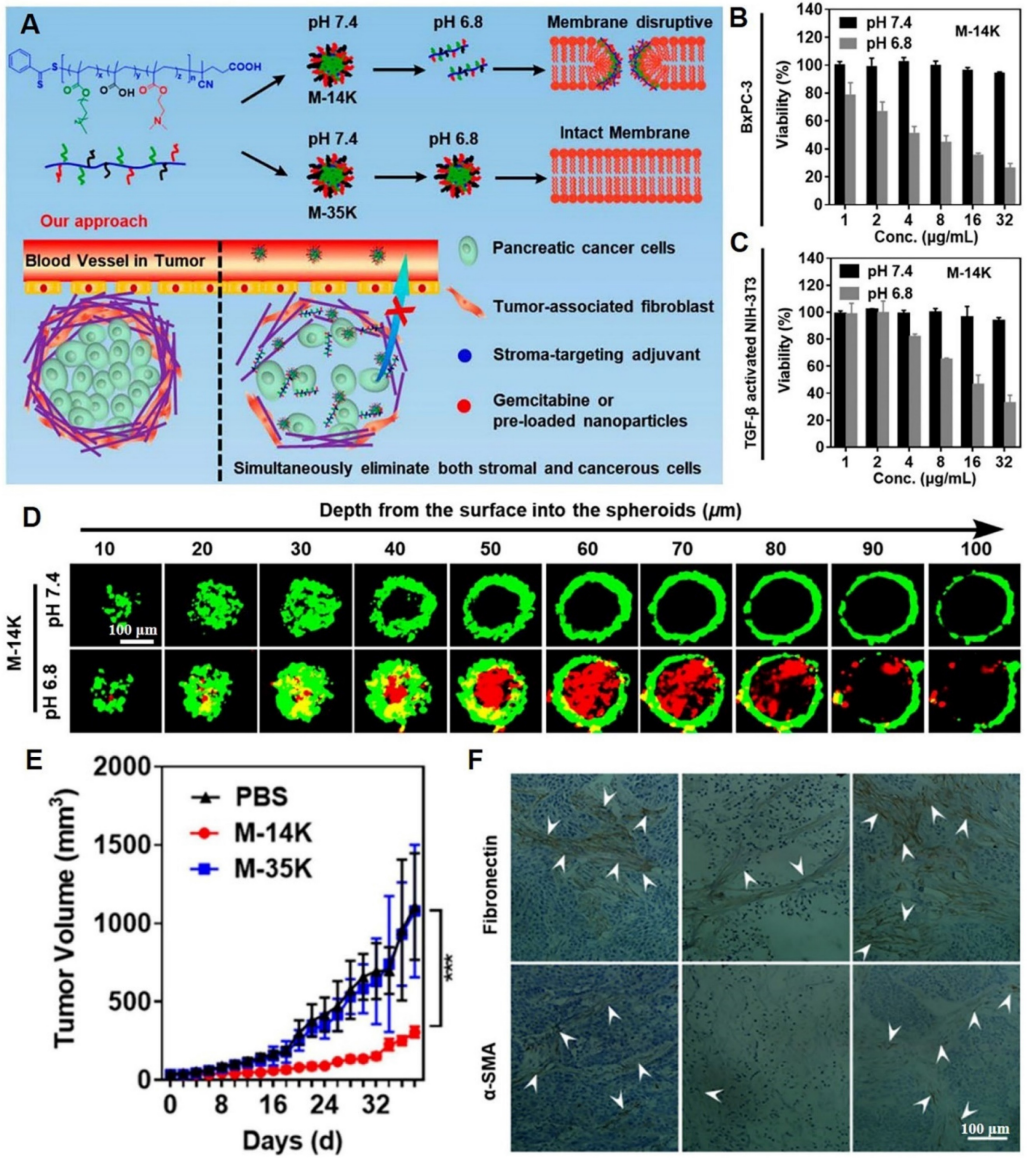
Membrane-disruptive nanotherapeutics for dissolving the dense extracellular matrix. (A) Schematic illustration on acid-activatable and membrane-disruptive nanomicelles (M-14K) for simultaneously eliminating cancerous and stromal cells. The viability assays of (B) BxPC-3 and (C) activated-fibroblasts NIH-3T3 cells. (D) The images of three-dimensional BxPC-3@NIH-3T3 spheroids with a shell of fibroblast cells (green) and a core of cancerous cells after M-14K treatment. Propidium iodide (red) was used to stain dead cells. (E) Tumor volume during therapy. (F) The immunohistochemistry-stained fibronectin and α-SMA of tumor tissues. (Adapted with permission from Ref. 98. Copyright 2021 American Chemical Society)

In another study, acid-activated NPs composed of membrane-disruptive molecules were developed. These NPs consisted of cationic and amphipathic motifs and were capable of distinguishing between the acidic TME and normal physiological pH. They exhibited acid-induced dissociation, thereby indiscriminately damaging cell membrane integrity of both stromal and cancer cells (**Figure 5A**) 98. Acid-sensitive nanomaterials (M-14K) exhibited dose-dependent cytotoxic effects against both BxPC-3 pancreatic carcinoma cells and NIH-3T3 fibroblast cells (**Figure 5B-C**). As confirmed by the core-shell structured BxPC-3@NIH-3T3 spheroids model* in vitro*, M-14K significantly penetrated the stromal barrier and eradicated hiding carcinoma cells via acid-activatable effects (**Figure 5D**). Scanning electron microscopy showed that BxPC-3 cell surfaces had abundant cracks and pores at pH 6.8, while being smooth and intact at pH 7.4. M-14K treatment significantly delayed tumor growth in BxPC-3 xenograft mouse models, while significantly remodeling the stroma and reducing fibronectin as well as α-SMA levels (**Figure 5E-F**). A decrease in the number of tumor-associated fibroblasts turned the tight barrier into a loose porous medium that facilitated drug penetration and swept away the shielded cancer cells. In biosafety research, M-14K did not exert any effects on body weights of mice. The H&E-stained slice images of major organs confirmed the biosafety of M-14K.

**Figure 6:**
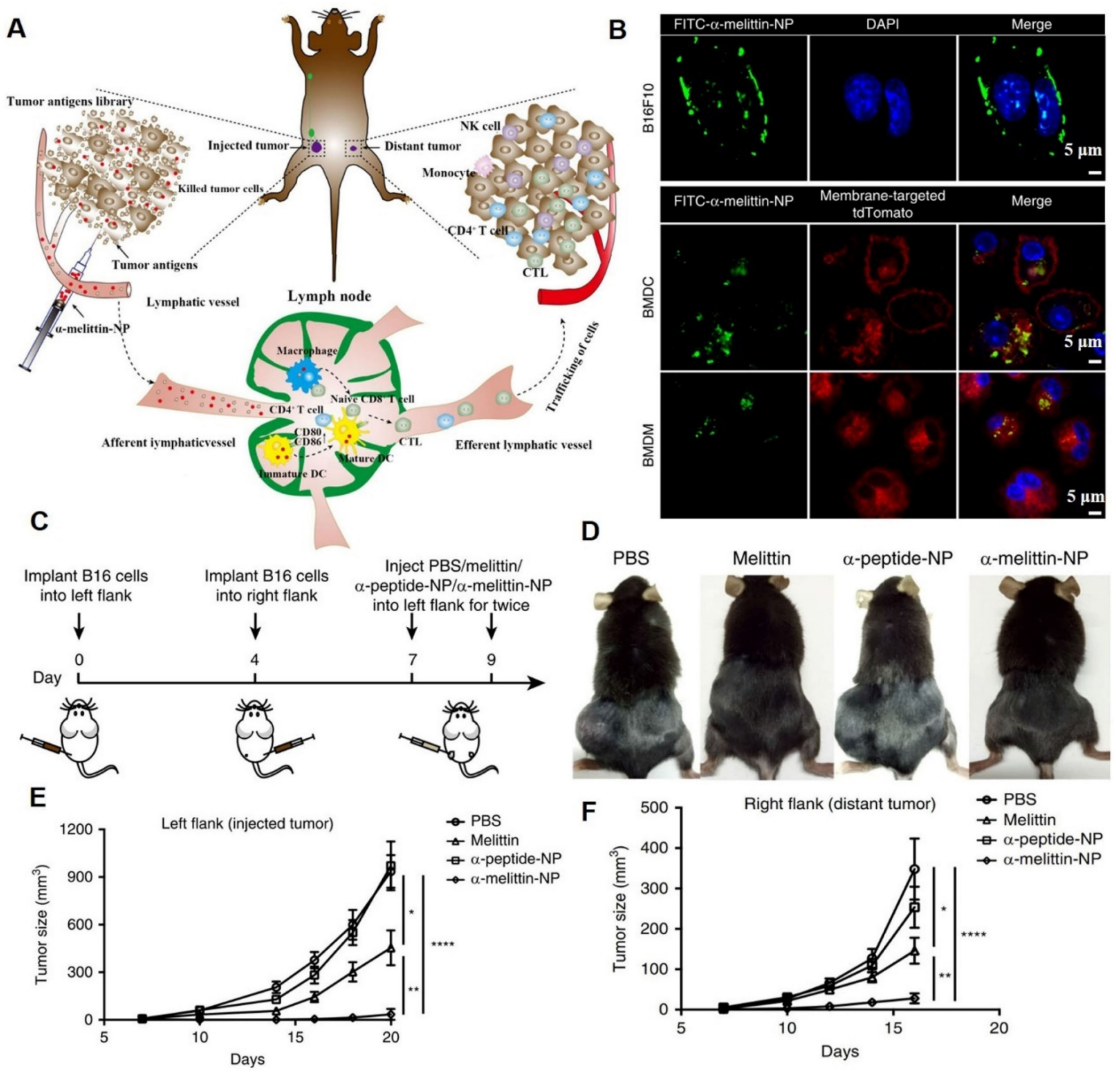
Melittin-lipid NPs induced tumor cell membranolytic effect. (A) Schematic illustration of the antitumor mechanism of α-melittin-NPs. The membranolytic effect of α-melittin-NPs could kill tumor cells and promote the release of whole tumor-cell antigens. (B) Representative immunofluorescence imaging of cellular binding of FITC-α-melittin-NPs to B16F10 cells and antigen-presenting cells (BMDCs and BMDMs). BMDCs and BMDMs were isolated from mT/mG mice that express a strong red fluorescence protein (tdTomato) in the membrane systems (plasma membrane, lysosome) of all cell types. Blue: DAPI, green: FITC-α-melittin-NPs, red: membrane-targeted tdTomato. (C) Treatment scheme. (D) Representative pictures of mice treated with melittin, α-peptide-NPs, or α-melittin-NPs. Tumor growth of the injected tumor (E) and distant tumor (F). (Adapted with permission from Ref. 12. Copyright 2020 Springer Nature)

Membrane-lytic peptides are efficient membrane-disruptive molecules that are of significant interest in medical science 13. Melittin, an antimicrobial peptide, was proposed for anticancer treatment in the 2000s. The cationic charge and amphipathic properties of melittin enable it to interact with and disrupt biological membranes 14. To overcome the limitations of melittin in antitumor applications (such as rapid degradation/clearance, poor tumor accumulation, and nonspecific hemolysis), Yu et al. designed melittin-lipid nanoparticles (α-melittin-NPs) for cancer therapy (**Figure 6A**) 12. Confocal imaging data showed that after incubation for 3 h, FITC-α-melittin-NPs exhibited a good affinity with cell membranes (**Figure 6B**). The tumor growth curve showed that α-melittin-NPs significantly suppressed tumor growth on the left flank (injected tumor) and right flank (distant tumor). At 20 days post left tumor inoculation, compared with the phosphate buffer solution (PBS) group, mice treated with α-melittin-NPs showed a 95% decrease in injected tumor size and a 92% decrease in distant tumor size (**Figure 6C-F**). Other melittin-containing nanodrugs also exhibited strong membranolytic effects and superior antitumor effects 13, 14.

The cell membrane is an armor of tumor cells. Once it is breached, tumor cells collapse. Ruptured cell membranes are difficult to repair because — unlike proteins or nucleic acids — they are not encoded by the genome or plasmids 99. Compared to conventional inert polymers that serve as drug delivery vehicles, membranolytic molecules composed solely of active ingredients target and dissolve the membrane lipid bilayer skeleton. This is in stark contrast with traditional chemotherapeutics, which target specific intracellular substances or metabolic pathways that can be repaired by transcriptional or other types of compensation. Membranolytic actions are also effective against dormant cancer 100 and MDR cells. Non-selective membranolytic effects and stroma membrane lysis effects exert strong and irreparable outcomes on normal cells. Studies should investigate the influence of membrane lipid therapeutic strategies on nonmalignant cell membranes to facilitate clinical translation. Lytic effects on normal cells can be reduced by delicately tuning structural parameters or developing stimuli-responsive moieties.

### Lipid raft immobilization tumor nanotherapeutics

**Figure 7:**
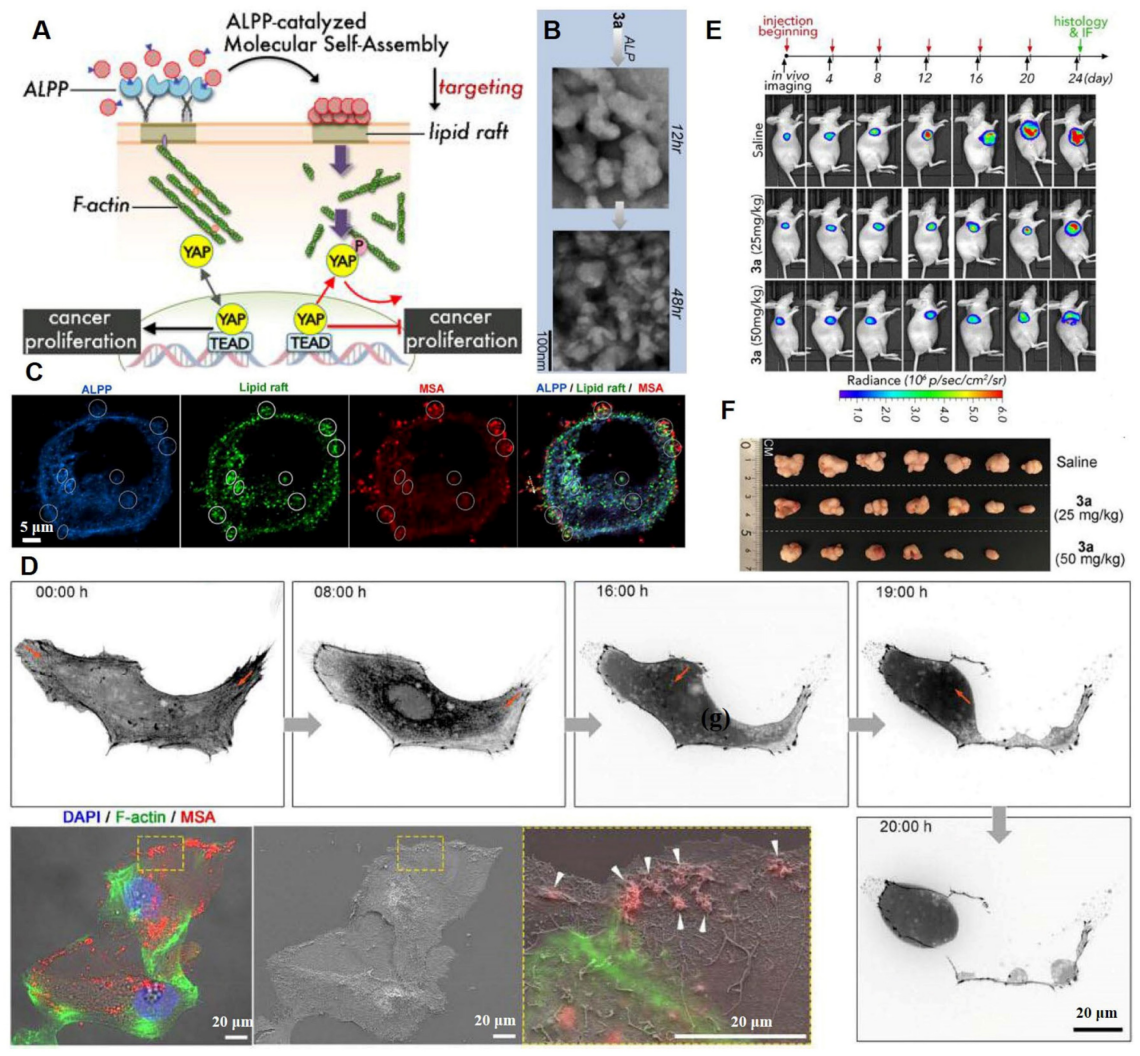
Lipid raft immobilization and signaling pathway interference by the precursor molecules. (A) Schematic representation of ALPP-guided and lipid-raft-targeted MSA inactivates Yes-associated protein via actin cytoskeleton disruption for antitumor. (B) ALP dephosphorylation of 3a (three hydrolysable groups) triggers MSA at different time scales. (C) Immunofluorescence staining of ALPP and lipid raft on SKOV3 cells upon 3a incubation. (D) Time-lapse images of abnormal cell motion with broken trailing tails upon 3a incubation and correlative light-electron microscopy analysis indicates interrupted actin dynamics by MSAs forming on the cell margin. (E) The SKOV3-Luc-tumor growth was monitored by bioluminescence detection before each injection. (F) Photographs of tumors at day 24. (Adapted with permission from Ref. 103. Copyright 2020 American Chemical Society)

The cytoskeleton is tightly tethered to its binding partners in lipid rafts. Lipid rafts and the underlying cytoskeleton are involved in transduction of many tumor signaling pathways 101. Regulation of signaling pathways by P2Y2 nucleotide receptors in glioma cells depends on cytoskeleton dynamics, altering stress fiber assembly and thereby affecting tumor cell adhesion as well as migration 102. F-actin/cytoskeleton reorganization can be induced by intervening plasma membrane lipid raft stiffness and rigidity, which promotes the phosphorylation of transcription coactivators and tumor cell apoptosis 103. A ruthenium-complex-peptide precursor molecule was designed to generate molecular self-assembly (MSA) nanostructures under the guidance of alkaline phosphatase (ALPP), an ovarian cancer biomarker 103. Extension of MSA into lipid rafts led to formation of stable and solid nanobiointerfaces, resulting in actin cytoskeleton recombination and inactivation of oncogenic Yes-associated proteins (**Figure 7A**). The dephosphorylation of precursor 3a induced MSA contributed to formation of irregular and denser aggregates (**Figure 7B**). Co-localization of ALPP, MSAs, and large lipid raft clusters on SKOV3 cells indicated the lipid-raft-targeting abilities of these NPs **(Figure 7C)**. Moreover, during movement, SKOV3 cells exhibited disrupted tail retraction, which was caused by interrupted actin dynamics and broken trailing tails. This was because the MSAs solidified the lipid rafts **(Figure 7D)**. The MSAs exhibited the strongest cytotoxic effects against cancer cells with elevated ALPP expressions. Finally, after the 24-day treatment period, 3a treatment induced dose-dependent SKOV3-Luc tumor suppression and 60% tumor reduction (50 mg/kg dose) **(Figure 7E-F)**. The H&E-stained slice images of major organs indicated the biosafety of the designed nanoplatforms, which were more effective against highly metastatic cancer cells.

### Energy-mediated plasma membrane perturbation tumor nanotherapeutics

External energy sources can enhance tumor cell membrane permeability, improving targeted drug transport efficiency and local tumor tissue damage. The common energy-mediated plasma membrane perturbation approaches involve ultrasound stimulation 104, electrical stimulation 105, and laser irradiation 106.

#### Ultrasound stimulation plasma membrane perturbation tumor nanotherapeutics

**Figure 8:**
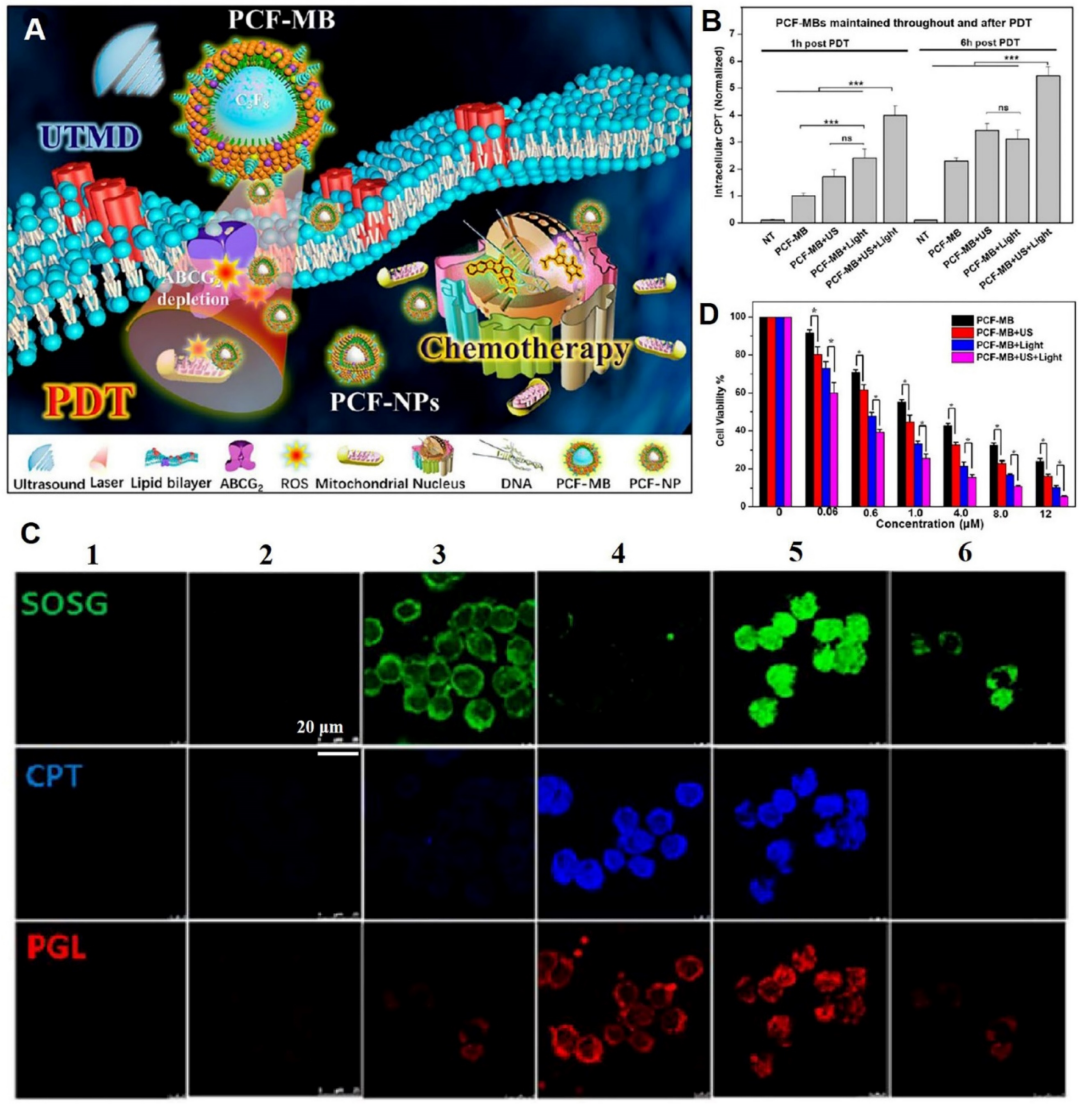
Ultrasound triggered sonoporation of membranes and conversion of microbubbles into NPs for reversing drug resistance. (A) Schematic illustration of ultrasound-triggered conversion of PCF-MBs into PCF-NPs mediated chemo-photodynamic therapy and PDT-induced ABCG_2_ depletion for overcoming MDR. (B) Intracellular CPT levels of PCF-MBs post-PDT. (C) Cell uptake (camptothecin (CPT), blue; PGL, red) and singlet oxygen generation (green) after different treatments (1: control, 2: PCF-MBs, 3: PCF-MBs+light, 4: PCF-MBs+ultrasound, 5: PCF-MBs+ultrasound+light, and 6: PGL-MBs+ultrasound+light). (D) Concentration-dependent cytotoxicity in HT-29 cells after different treatments at 72 h. (Adapted with permission from Ref. 110. Copyright 2018 American Chemical Society)

Ultrasound-derived energy can destroy biological barriers in a safe and selective manner. Ultrasound induces sonoporation, creating temporary pores in cell membranes to enhance cell endocytosis 107. Microbubbles, which are structurally similar to cell membranes, oscillate under ultrasound treatment 108. Low-intensity ultrasound produces microbubbles with stable cavitation, while high-intensity ultrasound produces microbubbles with inertial cavitation, which collapse to form sonophoresis. Membrane permeabilization can be achieved by stabilizing cavitation microbubbles, which enables small or macromolecular drugs to be transported across membranes 109. To improve MDR colorectal cancer treatment, Chen *et al*. proposed a combination strategy involving stable triplex porphyrin/camptothecin-floxuridine microbubbles (PCF-MBs) generated by cavitation of photosensitizer porphyrin-grafted lipids (PGLs), amphiphilic camptothecin-floxuridine (CF) conjugates, and solvents 110. The ultrasound-induced sonoporation of membranes and *in situ* conversion of PCF-MBs to PCF-NPs resulted in elevated accumulation of chemotherapeutics and photosensitizers in tumors. Photodynamics induced the depletion of the drug efflux transporter (ABC subfamily G member 2) on cell membranes and prolonged drug retention **(Figure 8A-B)**. After ultrasound exposure, uptake of PGL and CF drugs by HT-29 cells was increased, accompanied by elevated singlet oxygen generation under light irradiation **(Figure 8C).** Thus, significant concentration-dependent cytotoxic effects against MDR cells were achieved *in vitro* **(Figure 8D)**, and the synergistic photodynamic therapy (PDT) and chemotherapy resulted in a 90% tumor suppression rate *in vivo*. These findings show that application of ultrasound to enhance transmembrane transport of nanodrugs is a promising translational strategy for treatment of drug-resistant tumors. In biosafety research, H&E staining images of major organs and body weight curves showed that combined chemo-PDT treatment had no unacceptable toxic effects.

#### Electrical stimulation plasma membrane perturbation tumor nanotherapeutics

Applications of a short and continuous pulsed electric field (with energy) induces cell membrane perturbation. It also produces transient pores in cell membranes, a process known as electroporation 111. Under treatment with an electric field, the energy required for water molecules to penetrate the lipid bilayer is reduced. Water can rapidly enter the lipid layer of cell membranes, causing the adjacent lipids and their polar head groups to reorient. Such effects result in generation of pores within cell membranes 112. The pores enhance cell membrane permeability. The NPs can reach tumor sites via enhanced permeability and retention (EPR) effects. However, blood vessels, cell membranes, and cell-matrix spaces block the delivery efficiency of nanosystems. Therefore, disruption of physiological barriers formed by membranes is a potential tumor treatment strategy. A study investigating the effects of electroporation on tumor vasculature and microenvironment showed that electroporation can modulate the above barriers that prevent NPs from penetrating the tumor 105. That is, it can create voids in the vasculature, cell membrane, and cell-matrix spaces; form intercellular spaces between endothelial cells, which change with electric field magnitude, and allow the NPs to penetrate deeper into the tumor and accumulate. These effects result in strong antitumor outcomes. Lee et al reported that by combining single-walled carbon nanotubes (SWNT) with low-voltage (LV) electrical stimulation, biomolecule delivery could be effectively enhanced through reversible electroporation 113. Clear pore formation in the cell membrane is observed due to LV pulse electrical stimulation amplified by SWNT. In cancer treatment, both SWNT+LV pulse treatment followed by the injection of liposomal DOX or SWNT/DOX+LV pulse treatment can increase tumor inhibition and delay tumor growth. These studies provided a theoretical basis for treatment of tumors via electrical stimulation-mediated cell membrane alteration.

#### Laser irradiation plasma membrane perturbation tumor nanotherapeutics

Phototherapy is a prospective cancer therapeutic modality with high selectivity, low invasiveness and ease of remote control. Under laser irradiation, photosensitizers generate ROS, which target the double bonds of lipids to generate lipid peroxides 114. Massive oxidation of PC in cell membranes damages lipid bilayer stability, promoting the generation of membrane voids and improving membrane penetrance 115. A study in 2018 reported that plasma membrane-anchored photosensitive NPs, consisting of protoporphyrin IX (PpIX, a photosensitizer) conjugated to PEG-cholesterol polymer (Chol-PEG-PpIX), exhibited lipid raft-responsive and light-controllable cytoplasmic transport effects 106. The cholesterol fraction acts as a hydrophobic anchor to the cell membrane that affords lipid raft-mediated endocytosis, bypassing endosomal/lysosomal entrapment, which promotes PpIX binding to cell surfaces. Upon laser irradiation, PpIX can effectively result in lipid peroxidation in the plasma membrane, thereby disrupting membrane permeability and allowing rapid influx of extracellular NPs. To weaken the non-specific binding ability *in vivo*, Chol-PEG-PpIX has been anchored on liposomes for systemic delivery. The PEG imparts stealth properties with a long circulation time, achieving superior tumor ablation by PDT. Therefore, light-driven cell membrane perturbation is a promising strategy for improving transmembrane efficiency of nanosystems.

## Summary and future perspectives

Cancers are a result of genetic mutations that cause epigenetic changes, leading to abnormal cell behaviors. For instance, efforts to suppress ovarian cancer metastasis have focused on genetic and epigenetic targeting. Cancer cells exhibit significant differences from normal cells in terms of their lipid components, protein expressions, morphology, and biological properties of plasma membranes. These differences are essential for invasion, immune escape, and metastasis. Therefore, a novel therapeutic strategy called “membrane lipid perturbing tumor therapy”, which aims at interfering with membrane-related cell proliferation, motility, adhesion, signal transduction, and immunity, has attracted a great deal of attention. Various membrane components have emerged as treatment targets (**Table 2**). This has increased research on membrane lipid perturbation for tumor therapy, and on expression patterns of membrane phospholipids as well as their receptors in several cancers. To expand the impact of membrane lipid perturbation in tumor therapy, this review first delineated the association between plasma membrane characteristics and tumor signaling, metastasis, adhesion, and drug resistance. Existing therapeutic strategies for nanotechnology-based cell membrane disruption were highlighted, revealing the innate advantages of nanotechnology in membrane lipid perturbation for tumor therapy. Nanotechnologies can enable specific aggregation of plasma membrane-interfering molecules in tumor cells via EPR effects and incorporate multiple membrane-interfering molecules with different mechanisms into one single drug delivery system for synergistic treatment. In general, nanotechnology enhances the flexibility and versatility of membrane lipid perturbation during tumor therapy and is expected to improve the specificity and effectiveness of this treatment approach.

Membrane lipid perturbing tumor therapy is an emerging field. More cell membrane-based tumor treatment strategies are yet to be developed. The novel lipid modulation strategies presented herein can inhibit tumorigenesis and progression. These strategies include altering plasma membrane fluidity via nanodrug-mediated regulation of cholesterol contents, unsaturated to saturated lipid ratio, and long- to short-chain ceramide ratio, thereby suppressing tumor cell metastasis, drug resistance, and plasma membrane-mediated signaling. Additionally, reprogramming lipid metabolism can disturb lipid reservoirs in cancer cells and deregulate the most important lipid pathway, i.e., the FA synthesis pathway. Agarwala *et al.* reported the relevant targets for disrupting FA synthesis 8, which can inform on designing of nanodrugs that interfere with FA synthesis and retard membrane lipid synthesis in tumor cells. Identification of novel targets and prognostic biomarkers for tumors is also warranted. This review provides insights on cancer lipidome and related membrane properties. These factors can be attributed to various cellular functions, which are modulated by lipids and associated membrane parameters. Lipid mobility, lateral organization, and kinetics regulate physiological membrane functions. Therefore, lipid molecules are key regulators of cancer progression, and there is a growing trend for therapeutic strategies targeting lipid-associated processes (including lipid synthesis, metabolism, localization, and transport). Studies on correlations between lipidome changes and membrane biophysical properties are becoming increasingly popular in cancer diagnosis. Construction of nanoplatforms that modulate biophysical characteristics of the plasma membrane or interfere with lipid metabolism will achieve antitumor treatment.

Despite the advances in plasma membrane lipid perturbing tumor nanotherapeutics, clinical translation is limited by various challenges. First, most of the membrane lipid perturbing tumor nanotherapeutics discussed in this review focus on curative efficacy rather than safety. Some membranolytic materials are extremely toxic to normal cells. There is a need to explore the effects of membranolytic nanoplatforms on normal cell membranes. Moreover, there is a need to improve the targeting abilities of these nanodrugs, and their organismal safety warrants greater scrutiny. Second, membrane lipid perturbation strategies can be customized for different types and stages of cancers to achieve optimal efficacy and minimal side effects. For instance, breast cancer cell plasma membranes exhibit high cholesterol expressions. Thus, cholesterol-modulating nanotherapeutics are more effective in breast cancer. Lipid profiles of specific tumor membranes should be investigated before selecting the appropriate treatment schemes. Third, effectiveness of these formulations has only been tested in small animals, validation should be conducted in bigger animals. Finally, membrane lipid perturbing nanoformulation designs should also account for industrial feasibility and economics, since a simple preparation process favors clinical translation. Some commonly used clinical nanocarriers, such as liposomes, PLGA, albumin nanoparticles, polymer micelles, can be preferentially selected for lipid-regulating drug delivery. Even though this paper highlights the potential of membrane lipid perturbing strategies in tumor therapy, cell membrane-based tumor therapies should not be restricted to this. In summary, with development and integration of multiple disciplines, tumor cell membranes will play more important roles in tumor treatment.

## Figures and Tables

**Table 1:** Membrane lipid therapeutic drugs in clinical trials for cancer treatment.

Condition or disease	Drug name	Phase	NCT number	Mechanism of action
Colorectal Cancer	Simvastatin	Phase 2	NCT02026583	Alteration in lipid raft cholesterol content
Breast Cancer	Simvastatin	Phase 2	NCT00334542	
Prostate Cancer	Simvastatin	Not applicable	NCT00572468	
Breast Cancer	Pitavastatin	Phase 2 Phase 3	NCT04705909	
Breast Cancer	Simvastatin Atorvastatin	Phase 3	NCT03971019	
Breast Cancer	Imipramine	Early Phase 1	NCT03122444	K-Ras mislocalization by altering the sphingomyelin and PS content
Children high-grade glioma solid tumor	2-Hydroxyoleic Acid	Phase 1 Phase 2	NCT04299191	
Glioma and other solid tumours	2-Hydroxyoleic Acid	Phase 1/2A	NCT01792310	
Non-squamous non-small cell lung cancer	Bavituximab	Phase 2	NCT01160601	Antitumor immunity by directly against PS; co-clustering of lipid rafts and death receptors
Malignant Gliomas	Perifosine	Phase 2	NCT00590954	
Colorectal Cancer	Resveratrol	Phase 1	NCT00920803	
Pancreatic Cancer	ABTL0812	Phase 1 Phase 2	NCT03417921	Increased rigidity of the endoplasmic reticulum membrane
Endometrial Cancer Squamous Non-Small Cell Lung Cancer	ABTL0812	Phase 1 Phase 2	NCT03366480	
Multiple Myeloma	ABC294640	Phase 1 Phase 2	NCT02757326	Disturb lipid metabolism enzymes

**Table 2:** The classification and mechanisms of plasma membrane lipid perturbing tumor nanotherapeutics.

Classification	Therapeutic mechanisms	Functional nanoplatforms	Cargoes	Cancer types	Ref.
Plasma membrane lipid peroxide accumulation	Lipid peroxide generation	Folate-PEG modified manganese doped mesoporous silica NPs	Dihydroartemisinin	Hepatocellular cancer and ovarian cancer	87
	Lipid peroxide generation	Hybrid metal organic framework decorated with polydopamine and PEG	Perfluoropentane	Hepatocellular cancer	116
	Lipid peroxide generation	Fe^3+^/tea polyphenol epigallocatechin gallate metal-phenolic networks	DOX	Lung cancer	117
	Lipid peroxide generation	cRGD-modified polydopamine-based nanoplatform	Fe^2+^ and β-lapachone	Melanoma	118
Plasma membrane cholesterol regulation	Cholesterol extraction from the cell membrane	CD44-receptor targetable methyl-β-cyclodextrin-modified hyaluronic acid-ceramide nano-assembly	Not available	Breast cancer	90
	Blocking ACAT-1-mediated cholesterol esterification	Human serum albumin	Avasimibe	Prostate and colon cancer	119
	Cholesterol depletion	Nanosphere and nanocapsules based on amphiphilic α- and β-cyclodextrin	Erlotinib	Lung and hepatocellular cancer	120
	Lipid rafts (cholesterol-rich domains) disruption to reverse EMT and repolarize TAM	TME-associated protease legumain sensitive peptide modified liposome	Simvastatin and PTX	Lung cancer	121
	Cholesterol consumption in lipid rafts and P-gp down-regulation	α-tocopherol PEG 2000 succinate modified PLGA NPs	DOX and simvastatin	Colon cancer	91
	Cascade catalytic consumption of cholesterol	Cholesterol oxidase immobilized on the metal organic framework NH2-MIL-88B and modified with chondroitin sulfate gel shell	DOX, cholesterol oxidase	Breast cancer	92
	Inhibition of cholesterol esterification in CD8^+^ T cells to enhance chemo-immunotherapy	Liposome modified with pH sensitive TH peptide	PTX and immunoadjuvant αGC	Melanoma	94
Plasma membrane structure disruption	Membrane disruption and mitochondria damage	Alginate hydrogel polymer	Gold NPs and cisplatin	Human nasopharyngeal cancer	122
	Enhanced apoptosis and necrosis* in vitro* by combined drug-loading	Aragonite calcium carbonate NPs	DOX and thymoquinone	Breast cancer	123
	Increased DOX cytotoxicity	γ-Fe_2_ O_3_ NPs stabilized with carboxymethylcellulose sodium	DOX	Breast Cancer	124
	Disruption of mitochondrial Ca^2+^ homeostasis	Calcium phosphate-doped hollow mesoporous copper sulfide	Curcumin	Breast cancer	125
	Membrane lysis followed by necrosis	Cationic diblock polycarbonates	Not available	Hepatocellular carcinoma	126
	A non-apoptotic mechanism with significant vacuolization and membrane disruption	Triblock copolymers of PEG, guanidinium-functionalized polycarbonate and polylactide	Not available	Breast cancer	127
	Membranolytic effect	A PEG block and pH-responsive membranolytic block that contains ionizable tertiary amine segments (ethylpiperidine) and hydrophobic segments	Not available	Pancreatic cancer, colon tumor, melanoma and lung metastasis	97
	Membranolytic effect	Self-assembling melittin-lipid nanoparticle	Not available	B16F10 melanoma	12
	Membranolytic effect	D-melittin micelles	Not available	Breast cancer and colon cancer	14
	Membranolytic effect	PEG-PLGA NPs	Membrane-Lytic Peptide	Triple-negative breast cancer	13
	Acid-activatable membrane disruption of both stromal and cancerous cells	Random copolymers of hexylmethacrylate, dimethyl aminoethylmethacrylate, and methacrylic acid	Not available	Pancreatic cancer	98
	Membrane disruption and enhanced cellular uptake of chemotherapeutics, antibiotics and the polymer	Quaternary ammonium-functionalized cationic polycarbonate	Not available	Liver cancer and drug-resistant breast cancer	128
	P-gp inhibition and increased plasma membrane fluidity	pH-sensitive polymeric micelles based on poly(2-ethyl-2-oxazoline)-poly(D,L-lactide)	PTX and HNK	Pulmonary metastasis from breast cancer	129
Lipid raft immobilization effect	Lipid rafts solidification and actin cytoskeleton reorganization	Ruthenium-complex-peptide precursor molecule	Not available	Ovarian cancer	103
Energy-mediated plasma membrane perturbation	PDT enhancement	PpIX-anchored liposomes	PpIX	Cervical cancer	106
	Electroporation	Radiolabeled liposomal nanoparticle	DOX	Prostate cancer	130
	Electroporation	Silica NPs	Ruthenium fluorescent complex/Cy7	Colon cancer and B-lymphoma	131
	Electroporation	Polyethylenimine NPs	BAS and tannic acid	Glioblastoma	132
	Electroporation	Single-walled carbon nanotubes	DOX	Colon cancer	113
	Electroporation	Dye-stabilized sorafenib NPs	Sorafenib	Colon cancer	105
	Ultrasound cavitation	Contrast agents Sonazoid®	Not available	Colon cancer	133
	Ultrasound enhancement	Lipid/PLGA hybrid microbubbles	DOX	Breast cancer	134
	Ultrasound enhancement	Porphyrin/camptothecin-floxuridine microbubbles	Camptothecin and floxuridine	Colon cancer	110
	Ultrasound enhancement	Trastuzumab microbubbles	Trastuzumab	HER2-Positive Gastric Cancer	135
	Ultrasound enhancement	Nanobubbles with a lipid shell	Indocyanine green and PTX	Prostate cancer	136
	Ultrasound enhancement	Dual-targeting cationic microbubbles conjugated with iRGD peptides and CCR2 antibodies	iRGD peptides and CCR2 antibody	Breast cancer	137

## Abbreviations

ACC: acetyl-CoA carboxylase
FA: fatty acid
NPs: nanoparticles
SL: sphingolipids
PS: phosphatidyl serines
PC: phosphatidyl cholines
PE: phosphatidyl ethanolamines
PI: phosphatidyl inositols
PG: phosphatidyl glycerol
CTLs: cytotoxic T lymphocytes
IS: immune synapse
LPA: Lysophosphatidic acid
TGF: transforming growth factor
EMT: epithelial-mesenchymal transition
PTX: paclitaxel
TAMs: tumor-associated macrophages
ATP: adenosine triphosphate
ABC: ATP-binding cassette
TME: tumor microenvironment
MDR: multi-drug resistant
DOX: doxorubicin
P-gp: P-glycoprotein
ROS: reactive oxygen species
GPx4: glutathione peroxidase 4
GSH: glutathione
PEG: polyethylene glycol
DHA: dihydroartemisinin
·OH: hydroxyl radicals
MbCD: methyl-β-cyclodextrin
HA: hyaluronic acid
PLGA: poly (lactic-glycolic acid)
ACAT-1: acyl-CoA cholesterol acyltransferase-1
PBS: phosphate buffer solution
MSA: molecular self-assembly
ALPP: alkaline phosphatase
PCF: porphyrin/camptothecin-floxuridine
MB: microbubbles
PGLs: porphyrin-grafted lipids
PDT: photodynamic therapy
EPR effect: enhanced permeability and retention effect
SWNT: single-walled carbon nanotubes
LV: low-voltage
PpIX: protoporphyrin IX

## References

[1] Holohan C, Van Schaeybroeck S, Longley DB, Johnston PG (2013). Cancer drug resistance: an evolving paradigm. Nat Rev Cancer.

[2] Ivanova PT, Cerda BA, Horn DM, Cohen JS, McLafferty FW, Brown HA (2001). Electrospray ionization mass spectrometry analysis of changes in phospholipids in RBL-2H3 mastocytoma cells during degranulation. Proc Natl Acad Sci U S A.

[3] Riedl S, Rinner B, Asslaber M, Schaider H, Walzer S, Novak A (2011). In search of a novel target - Phosphatidylserine exposed by non-apoptotic tumor cells and metastases of malignancies with poor treatment efficacy. Biochim Biophys Acta Biomembr.

[4] Escribá PV (2006). Membrane-lipid therapy: a new approach in molecular medicine. Trends Mol Med.

[5] Zalba S, Ten Hagen TL (2017). Cell membrane modulation as adjuvant in cancer therapy. Cancer Treat Rev.

[6] Oommen D, Dodd NJF, Yiannakis D, Moyeed R, Jha AN (2016). Linking genotoxicity and cytotoxicity with membrane fluidity: A comparative study in ovarian cancer cell lines following exposure to auranofin. Mutat Res Genet Toxicol Environ Mutagen.

[7] Cheng C, Geng F, Cheng X, Guo D (2018). Lipid metabolism reprogramming and its potential targets in cancer. Cancer Commun.

[8] Agarwala PK, Aneja R, Kapoor S (2022). Lipidomic landscape in cancer: Actionable insights for membrane-based therapy and diagnoses. Med Res Rev.

[9] Codini M, Garcia-Gil M, Albi E (2021). Cholesterol and sphingolipid enriched lipid rafts as therapeutic targets in cancer. Int J Mol Sci.

[10] Li HN, Sun JY, Zhu HY, Wu HX, Zhang H, Gu ZW (2021). Recent advances in development of dendriticpolymer-basednanomedicines for cancer diagnosis. Wiley Interdiscip Rev Nanomed Nanobiotechnol.

[11] Chi XQ, Liu K, Luo XJ, Yin ZY, Lin HY, Gao JH (2020). Recent advances of nanomedicines for liver cancer therapy. J Mater Chem B.

[12] Yu X, Dai YF, Zhao YF, Qi SH, Liu L, Lu LS (2020). Melittin-lipid nanoparticles target to lymph nodes and elicit a systemic anti-tumor immune response. Nat Commun.

[13] Chen CH, Liu YH, Eskandari A, Ghimire J, Lin LCW, Fang ZS (2022). Integrated design of a membrane-lytic peptide-based intravenous nanotherapeutic suppresses triple-negative breast cancer. Adv Sci.

[14] Lv SX, Sylvestre M, Song KF, Pun SH (2021). Development of D-melittin polymeric nanoparticles for anti-cancer treatment. Biomaterials.

[15] Lin X, Lin X (2021). Surface ligand rigidity modulates lipid raft affinity of ultra-small hydrophobic nanoparticles: insights from molecular dynamics simulations. Nanoscale.

[16] Preta G (2020). New insights into targeting membrane lipids for cancer therapy. Front Cell Dev Biol.

[17] Peetla C, Vijayaraghavalu S, Labhasetwar V (2013). Biophysics of cell membrane lipids in cancer drug resistance: Implications for drug transport and drug delivery with nanoparticles. Adv Drug Delivery Rev.

[18] Choromanska A, Chwilkowska A, Kulbacka J, Baczynska D, Rembialkowska N, Szewczyk A (2021). Modifications of plasma membrane organization in cancer cells for targeted therapy. Molecules.

[19] Tan LTH, Chan KG, Pusparajah P, Lee WL, Chuah LH, Khan TM (2017). Targeting membrane lipid a potential cancer cure?. Front Pharmacol.

[20] Nelson ER, Chang C-y, McDonnell DP (2014). Cholesterol and breast cancer pathophysiology. Trends Endocrinol Metab.

[21] Lizardo DY, Parisi LR, Li NS, Atilla-Gokcumen GE (2018). Noncanonical roles of lipids in different cellular fates. Biochemistry.

[22] Lajoie P, Nabi IR (2010). Lipid rafts, caveolae, and their endocytosis. Int Rev Cell Mol Biol.

[23] Jacobson K, Mouritsen OG, Anderson RG (2007). Lipid rafts: at a crossroad between cell biology and physics. Nat Cell Biol.

[24] Simons K, Ehehalt R (2002). Cholesterol, lipid rafts, and disease. J Clin Invest.

[25] Torres M, Parets S, Fernandez-Diaz J, Beteta-Gobel R, Rodriguez-Lorca R, Roman R (2021). Lipids in pathophysiology and development of the membrane lipid therapy: new bioactive lipids. Membranes.

[26] Lingwood D, Simons K (2010). Lipid rafts as a membrane-organizing principle. Science.

[27] Huang BL, Song BL, Xu CQ (2020). Cholesterol metabolism in cancer: mechanisms and therapeutic opportunities. Nat Metab.

[28] Hryniewicz-Jankowska A, Augoff K, Biernatowska A, Podkalicka J, Sikorski AF (2014). Membrane rafts as a novel target in cancer therapy. Biochim Biophys Acta Rev Cancer.

[29] Hendrich AB, Michalak K (2003). Lipids as a target for drugs modulating multidrug resistance of cancer cells. Curr Drug Targets.

[30] Tekpli X, Holme JA, Sergent O, Lagadic-Gossmann D (2013). Role for membrane remodeling in cell death: implication for health and disease. Toxicology.

[31] Zhuang L, Lin J, Lu ML, Solomon KR, Freeman MR (2002). Cholesterol-rich lipid rafts mediate akt-regulated survival in prostate cancer cells. Cancer Res.

[32] Kajiwara K, Chen PK, Abe Y, Okuda S, Kon S, Adachi J (2022). Src activation in lipid rafts confers epithelial cells with invasive potential to escape from apical extrusion during cell competition. Curr Biol.

[33] Burgermeister E, Liscovitch M, Rocken C, Schmid RM, Ebert MPA (2008). Caveats of caveolin-1 in cancer progression. Cancer Lett.

[34] Sotgia F, Martinez-Outschoorn UE, Howell A, Pestell RG, Pavlides S, Lisanti MP (2012). Caveolin-1 and cancer metabolism in the tumor microenvironment: markers, models, and mechanisms. Annu Rev Pathol Mech Dis.

[35] Witkiewicz AK, Dasgupta A, Sammons S, Er O, Potoczek M, Guiles F (2010). Loss of stromal caveolin-1 expression predicts poor clinical outcome in triple negative and basal-like breast cancers. Cancer Biol Ther.

[36] Zhao XD, He YY, Gao J, Fan LF, Li ZH, Yang GF (2013). Caveolin-1 expression level in cancer associated fibroblasts predicts outcome in gastric cancer. PLoS ONE.

[37] Kreutzberger AJB, Ji M, Aaron J, Mihaljevic L, Urban S (2019). Rhomboid distorts lipids to break the viscosity-imposed speed limit of membrane diffusion. Science.

[38] Sheng R, Jung DJ, Silkov A, Kim H, Singaram I, Wang ZG (2016). Lipids Regulate Lck Protein Activity through Their Interactions with the Lck Src Homology 2 Domain. J Biol Chem.

[39] Lorent JH, Diaz-Rohrer B, Lin XB, Spring K, Gorfe AA, Levental KR (2017). Structural determinants and functional consequences of protein affinity for membrane rafts. Nat Commun.

[40] Cardone RA, Alfarouk KO, Elliott RL, Alqahtani SS, Ahmed SBM, Aljarbou AN (2019). The role of sodium hydrogen exchanger 1 in dysregulation of proton dynamics and reprogramming of cancer metabolism as a sequela. Int J Mol Sci.

[41] Omran Z, Rauch C (2014). Acid-mediated Lipinski's second rule: application to drug design and targeting in cancer. Eur Biophys J Biophy.

[42] Rauch C (2009). Toward a mechanical control of drug delivery. On the relationship between Lipinski's 2nd rule and cytosolic pH changes in doxorubicin resistance levels in cancer cells: a comparison to published data. Eur Biophys J Biophy.

[43] Bernardes N, Fialho AM (2018). Perturbing the dynamics and organization of cell membrane components: A new paradigm for cancer-targeted therapies. Int J Mol Sci.

[44] Kopecka J, Trouillas P, Gašparović AČ, Gazzano E, Assaraf YG, Riganti C (2020). Phospholipids and cholesterol: inducers of cancer multidrug resistance and therapeutic targets. Drug Resist Updat.

[45] Capitani N, Patrussi L, Baldari CT (2021). Nature vs. nurture: the two opposing behaviors of cytotoxic T lymphocytes in the tumor microenvironment. Int J Mol Sci.

[46] Basu R, Whitlock BM, Husson J, Le Floc'h A, Jin WY, Oyler-Yaniv A (2016). Cytotoxic T Cells Use Mechanical Force to Potentiate Target Cell Killing. Cell.

[47] Diz-Munoz A, Fletcher DA, Weiner OD (2013). Use the force: membrane tension as an organizer of cell shape and motility. Trends Cell Biol.

[48] Yu S, Wang L, Che D, Zhang M, Li M, Naito M (2022). Targeting CRABP-II overcomes pancreatic cancer drug resistance by reversing lipid raft cholesterol accumulation and AKT survival signaling. J Exp Clin Cancer Res.

[49] Pua TL, Wang F-q, Fishman DA (2009). Roles of LPA in ovarian cancer development and progression. Future Oncol.

[50] Kim E-K, Park J-M, Lim S, Choi JW, Kim HS, Seok H (2011). Activation of AMP-activated protein kinase is essential for lysophosphatidic acid-induced cell migration in ovarian cancer cells. J Biol Chem.

[51] Zuo W, Chen Y-G (2009). Specific activation of mitogen-activated protein kinase by transforming growth factor-β receptors in lipid rafts is required for epithelial cell plasticity. Mol Biol Cell.

[52] Jin HY, He Y, Zhao PF, Hu Y, Tao J, Chen J (2019). Targeting lipid metabolism to overcome EMT-associated drug resistance via integrin beta 3/FAK pathway and tumor-associated macrophage repolarization using legumain-activatable delivery. Theranostics.

[53] Yang Y-F, Chang Y-C, Jan Y-H, Yang C-J, Huang M-S, Hsiao M (2020). Squalene synthase promotes the invasion of lung cancer cells via the osteopontin/ERK pathway. Oncogenesis.

[54] Cheng XL, Smith JC (2019). Biological Membrane Organization and Cellular Signaling. Chem Rev.

[55] Sezgin E, Levental I, Mayor S, Eggeling C (2017). The mystery of membrane organization: composition, regulation and roles of lipid rafts. Nat Rev Mol Cell Biol.

[56] Braig S, Schmidt BUS, Stoiber K, Handel C, Mohn T, Werz O (2015). Pharmacological targeting of membrane rigidity: implications on cancer cell migration and invasion. New J Phys.

[57] Kaur J, Sanyal SN (2010). Alterations in membrane fluidity and dynamics in experimental colon cancer and its chemoprevention by diclofenac. Mol Cell Biochem.

[58] Sade A, Tuncay S, Cimen I, Severcan F, Banerjee S (2012). Celecoxib reduces fluidity and decreases metastatic potential of colon cancer cell lines irrespective of COX-2 expression. Biosci Rep.

[59] Subczynski WK, Wisniewska A (2000). Physical properties of lipid bilayer membranes: relevance to membrane biological functions. Acta Biochim Pol.

[60] Nicolson GL, Ash ME (2014). Lipid replacement therapy: a natural medicine approach to replacing damaged lipids in cellular membranes and organelles and restoring function. Biochim Biophys Acta Biomembr.

[61] Turk HF, Chapkin RS (2013). Membrane lipid raft organization is uniquely modified by n-3 polyunsaturated fatty acids. Prostaglandins Leukot Essent Fatty Acids.

[62] Yu Y, Skocaj M, Kreft ME, Resnik N, Veranic P, Franceschi P (2016). Comparative lipidomic study of urothelial cancer models: association with urothelial cancer cell invasiveness. Mol Biosyst.

[63] Huber F, Schnauss J, Ronicke S, Rauch P, Muller K, Futterer C (2013). Emergent complexity of the cytoskeleton: from single filaments to tissue. Adv Phys.

[64] Szachowicz-Petelska B, Sulkowski S, Figaszewski ZA (2007). Altered membrane free unsaturated fatty acid composition in human colorectal cancer tissue. Mol Cell Biochem.

[65] Yeung T, Grinstein S (2007). Lipid signaling and the modulation of surface charge during phagocytosis. Immunol Rev.

[66] Olivotto M, Arcangeli A, Carla M, Wanke E (1996). Electric fields at the plasma membrane level: A neglected element in the mechanisms of cell signalling. Bioessays.

[67] Szachowicz-Petelska B, Dobrzynska I, Skrodzka M, Darewicz B, Figaszewski ZA, Kudelski J (2013). Phospholipid composition and electric charge in healthy and cancerous parts of human kidneys. J Membr Biol.

[68] Goldenberg NM, Steinberg BE (2010). Surface charge: A key determinant of protein localization and function. Cancer Res.

[69] De M, Ghosh S, Sen T, Shadab M, Banerjee I, Basu S (2018). A Novel Therapeutic Strategy for Cancer Using Phosphatidylserine Targeting Stearylamine-Bearing Cationic Liposomes. Mol Ther Nucleic Acids.

[70] Ayesa U, Gray BD, Pak KY, Chong PLG (2017). Liposomes containing lipid-soluble Zn(II)-Bis-dipicolylamine derivatives show potential to be targeted to phosphatidylserine on the surface of cancer cells. Mol Pharm.

[71] Niero EL, Rocha-Sales B, Lauand C, Cortez BA, de Souza MM, Rezende-Teixeira P (2014). The multiple facets of drug resistance: one history, different approaches. J Exp Clin Cancer Res.

[72] Cohen AW, Hnasko R, Schubert W, Lisanti MP (2004). Role of caveolae and caveolins in health and disease. Physiol Rev.

[73] Eytan GD, Regev R, Oren G, Assaraf YG (1996). The role of passive transbilayer drug movement in multidrug resistance and its modulation. J Biol Chem.

[74] Preetha A, Banerjee R, Huilgol N (2007). Tensiometric profiles and their modulation by cholesterol: implications in cervical cancer. Cancer Invest.

[75] Yun U-J, Lee J-H, Shim J, Yoon K, Goh S-H, Yi EH (2019). Anti-cancer effect of doxorubicin is mediated by downregulation of HMG-Co A reductase via inhibition of EGFR/Src pathway. Lab Invest.

[76] Gu L, Saha ST, Thomas J, Kaur M (2019). Targeting cellular cholesterol for anticancer therapy. The FEBS journal.

[77] Subramanian N, Schumann-Gillett A, Mark AE, O'Mara ML (2016). Understanding the accumulation of P-glycoprotein substrates within cells: The effect of cholesterol on membrane partitioning. Biochim Biophys Acta Biomembr.

[78] Raghavan V, Vijayaraghavalu S, Peetla C, Yamada M, Morisada M, Labhasetwar V (2015). Sustained epigenetic drug delivery depletes cholesterol-sphingomyelin rafts from resistant breast cancer cells, influencing biophysical characteristics of membrane lipids. Langmuir.

[79] Liu Y, Fu YL, Hu XX, Chen S, Miao JB, Wang Y (2020). Caveolin-1 knockdown increases the therapeutic sensitivity of lung cancer to cisplatin-induced apoptosis by repressing Parkin-related mitophagy and activating the ROCK1 pathway. J Cell Physiol.

[80] Li ZY, Wang N, Huang CX, Bao YH, Jiang YQ, Zhu GT (2017). Downregulation of caveolin-1 increases the sensitivity of drug-resistant colorectal cancer HCT116 cells to 5-fluorouracil. Oncol Lett.

[81] Zou W, Ma X, Hua W, Chen B, Cai G (2015). Caveolin-1 mediates chemoresistance in cisplatin-resistant ovarian cancer cells by targeting apoptosis through the Notch-1/Akt/NF-κB pathway. Oncol Rep.

[82] Yang CPH, Galbiati F, Volonte D, Horwitz SB, Lisanti MP (1998). Upregulation of caveolin-1 and caveolae organelles in Taxol-resistant A549 cells. FEBS Lett.

[83] Sung M, Tan XZ, Lu BW, Golas J, Hosselet C, Wang F (2018). Caveolae-mediated endocytosis as a novel mechanism of resistance to trastuzumab emtansine (T-DM1). Mol Cancer Ther.

[84] Van der Paal J, Neyts EC, Verlackt CC, Bogaerts A (2016). Effect of lipid peroxidation on membrane permeability of cancer and normal cells subjected to oxidative stress. Chem Sci.

[85] Fei W, Zhang Y, Ye Y, Li C, Yao Y, Zhang M (2020). Bioactive metal-containing nanomaterials for ferroptotic cancer therapy. J Mater Chem B.

[86] Tang HX, Chen DF, Li CQ, Zheng CH, Wu XD, Zhang Y (2019). Dual GSH-exhausting sorafenib loaded manganese-silica nanodrugs for inducing the ferroptosis of hepatocellular carcinoma cells. Int J Pharm.

[87] Fei WD, Chen DF, Tang HX, Li CQ, Zheng WZ, Chen FY (2020). Targeted GSH-exhausting and hydroxyl radical self-producing manganese-silica nanomissiles for MRI guided ferroptotic cancer therapy. Nanoscale.

[88] Hancock JF (2006). Lipid rafts: contentious only from simplistic standpoints. Nat Rev Mol Cell Biol.

[89] Huo HR, Guo XM, Hong SY, Jiang MR, Liu XY, Liao K (2003). Lipid rafts/caveolae are essential for insulin-like growth factor-1 receptor signaling during 3T3-L1 preadipocyte differentiation induction. J Biol Chem.

[90] Lee SY, Ko SH, Shim JS, Kim DD, Cho HJ (2018). Tumor Targeting and Lipid Rafts Disrupting Hyaluronic Acid-Cyclodextrin-Based Nanoassembled Structure for Cancer Therapy. ACS Appl Mater Interfaces.

[91] Du B, Zhu W, Yu L, Wang Y, Zheng M, Huang J (2021). TPGS2k-PLGA composite nanoparticles by depleting lipid rafts in colon cancer cells for overcoming drug resistance. Nanomedicine.

[92] Du B, Zheng M, Ma H, Huang J, Jiao Q, Bai Y (2022). Nanozyme-natural enzymes cascade catalyze cholesterol consumption and reverse cancer multidrug resistance. J Nanobiotechnol.

[93] Molnar E, Swamy M, Holzer M, Beck-Garcia K, Worch R, Thiele C (2012). Cholesterol and Sphingomyelin Drive Ligand-independent T-cell Antigen Receptor Nanoclustering. J Biol Chem.

[94] Li M, Yang Y, Wei J, Cun X, Lu Z, Qiu Y (2018). Enhanced chemo-immunotherapy against melanoma by inhibition of cholesterol esterification in CD8+ T cells. Nanomedicine.

[95] Rink JS, Lin AY, McMahon KM, Calvert AE, Yang S, Taxter T (2020). Targeted reduction of cholesterol uptake in cholesterol-addicted lymphoma cells blocks turnover of oxidized lipids to cause ferroptosis. J Biol Chem.

[96] Kuzu OF, Noory MA, Robertson GP (2016). The Role of Cholesterol in Cancer. Cancer Res.

[97] Liu M, Huang L, Zhang W, Wang X, Geng Y, Zhang Y (2022). A transistor-like pH-sensitive nanodetergent for selective cancer therapy. Nat Nanotechnol.

[98] Fan F, Jin LJ, Yang LH (2021). pH-Sensitive Nanoparticles Composed Solely of Membrane-Disruptive Macromolecules for Treating Pancreatic Cancer. ACS Appl Mater Interfaces.

[99] Gaudet RG, Zhu SW, Halder A, Kim BH, Bradfield CJ, Huang S (2021). A human apolipoprotein L with detergent-like activity kills intracellular pathogens. Science.

[100] Takahashi H, Yumoto K, Yasuhara K, Nadres ET, Kikuchi Y, Taichman RS (2019). Anticancer polymers designed for killing dormant prostate cancer cells. Sci Rep.

[101] Head BP, Patel HH, Insel PA (2014). Interaction of membrane/lipid rafts with the cytoskeleton: impact on signaling and function: membrane/lipid rafts, mediators of cytoskeletal arrangement and cell signaling. Biochim Biophys Acta Biomembr.

[102] Kłopocka W, Korczyński J, Pomorski P (2013). Cytoskeleton and Nucleotide Signaling in Glioma C6 Cells. Adv Exp Med Biol.

[103] Li GY, Hu XW, Nie PP, Mang DZ, Jiao S, Zhang SJ (2021). Lipid-Raft-Targeted Molecular Self-Assembly Inactivates YAP to Treat Ovarian Cancer. Nano Lett.

[104] Zhang DY, Dmello C, Chen L, Arrieta VA, Gonzalez-Buendia E, Kane JR (2020). Ultrasound-mediated Delivery of Paclitaxel for Glioma: A Comparative Study of Distribution, Toxicity, and Efficacy of Albumin-bound Versus Cremophor Formulations. Clin Cancer Res.

[105] Kodama H, Shamay Y, Kimura Y, Shah J, Solomon SB, Heller D (2019). Electroporation-induced changes in tumor vasculature and microenvironment can promote the delivery and increase the efficacy of sorafenib nanoparticles. Bioelectrochemistry.

[106] Jia HR, Zhu YX, Xu KF, Liu X, Wu FG (2018). Plasma membrane-anchorable photosensitizing nanomicelles for lipid raft-responsive and light-controllable intracellular drug delivery. J Control Release.

[107] Lentacker I, De Cock I, Deckers R, De Smedt SC, Moonen CT (2014). Understanding ultrasound induced sonoporation: definitions and underlying mechanisms. Adv Drug Deliv Rev.

[108] Du M, Chen Z, Chen Y, Li Y (2019). Ultrasound-Targeted Delivery Technology: A Novel Strategy for Tumor- Targeted Therapy. Curr Drug Targets.

[109] van Wamel A, Kooiman K, Harteveld M, Emmer M, ten Cate FJ, Versluis M (2006). Vibrating microbubbles poking individual cells: Drug transfer into cells via sonoporation. J Control Release.

[110] Chen M, Liang XL, Gao C, Zhao RR, Zhang NS, Wang SM (2018). Ultrasound triggered conversion of porphyrin/camptothecin-fluoroxyuridine triad microbubbles into nanoparticles overcomes multidrug resistance in colorectal cancer. ACS Nano.

[111] Kumar P, Nagarajan A, Uchil PD (2019). Electroporation. Cold Spring Harb Protoc. 2019.

[112] Yarmush ML, Golberg A, Serša G, Kotnik T, Miklavčič D (2014). Electroporation-based technologies for medicine: principles, applications, and challenges. Annu Rev Biomed Eng.

[113] Lee PC, Peng CL, Shieh MJ (2016). Combining the single-walled carbon nanotubes with low voltage electrical stimulation to improve accumulation of nanomedicines in tumor for effective cancer therapy. J Control Release.

[114] Itri R, Junqueira HC, Mertins O, Baptista MS (2014). Membrane changes under oxidative stress: the impact of oxidized lipids. Biophys Rev.

[115] Cwiklik L, Jungwirth P (2010). Massive oxidation of phospholipid membranes leads to pore creation and bilayer disintegration. Chem Phys Lett.

[116] He HZ, Du LH, Guo HL, An YC, Lu LJ, Chen YL (2020). Redox Responsive Metal Organic Framework Nanoparticles Induces Ferroptosis for Cancer Therapy. Small.

[117] Mu M, Wang Y, Zhao S, Li X, Fan R, Mei L (2020). Engineering a pH/Glutathione-Responsive Tea Polyphenol Nanodevice as an Apoptosis/Ferroptosis-Inducing Agent. ACS Applied Bio Materials.

[118] Xue CC, Li MH, Liu CH, Li YN, Fei Y, Hu Y (2021). NIR-Actuated Remote Activation of Ferroptosis in Target Tumor Cells through a Photothermally Responsive Iron-Chelated Biopolymer Nanoplatform. Angew Chem Int Ed Engl.

[119] Lee SS-Y, Li J, Tai JN, Ratliff TL, Park K, Cheng J-X (2015). Avasimibe encapsulated in human serum albumin blocks cholesterol esterification for selective cancer treatment. ACS Nano.

[120] Varan G, Akkın S, Demirtürk N, Benito JM, Bilensoy E (2021). Erlotinib entrapped in cholesterol-depleting cyclodextrin nanoparticles shows improved antitumoral efficacy in 3D spheroid tumors of the lung and the liver. J Drug Target.

[121] Jin H, He Y, Zhao P, Hu Y, Tao J, Chen J (2019). Targeting lipid metabolism to overcome EMT-associated drug resistance via integrin β3/FAK pathway and tumor-associated macrophage repolarization using legumain-activatable delivery. Theranostics.

[122] Alamzadeh Z, Beik J, Pirhajati Mahabadi V, Abbasian Ardekani A, Ghader A, Kamrava SK (2019). Ultrastructural and optical characteristics of cancer cells treated by a nanotechnology based chemo-photothermal therapy method. J Photochem Photobiol B.

[123] Ibiyeye KM, Nordin N, Ajat M, Zuki ABZ (2019). Ultrastructural changes and antitumor effects of doxorubicin/thymoquinone-loaded CaCO3 nanoparticles on breast cancer cell line. Front Oncol.

[124] Lungu II, Nistorescu S Badea MA, Petre AM Udrea AM, Banici AM et al (2020). Doxorubicin-Conjugated Iron Oxide Nanoparticles Synthesized by Laser Pyrolysis: In Vitro Study on Human Breast Cancer Cells. Polymers.

[125] Xu L, Tong G, Song Q, Zhu C, Zhang H, Shi J (2018). Enhanced intracellular Ca(2+) nanogenerator for tumor-specific synergistic therapy via disruption of mitochondrial Ca(2+) homeostasis and photothermal therapy. ACS Nano.

[126] Park NH, Cheng W, Lai F, Yang C, Florez de Sessions P, Periaswamy B (2018). Addressing Drug Resistance in Cancer with Macromolecular Chemotherapeutic Agents. J Am Chem Soc.

[127] Zhong G, Yang C, Liu S, Zheng Y, Lou W, Teo JY (2019). Polymers with distinctive anticancer mechanism that kills MDR cancer cells and inhibits tumor metastasis. Biomaterials.

[128] Zheng Y, Kng J, Yang C, Hedrick JL, Yang YY (2021). Cationic polymer synergizing with chemotherapeutics and re-purposing antibiotics against cancer cells. Biomater Sci.

[129] Wang Z, Li X, Wang D, Zou Y, Qu X, He C (2017). Concurrently suppressing multidrug resistance and metastasis of breast cancer by co-delivery of paclitaxel and honokiol with pH-sensitive polymeric micelles. Acta Biomater.

[130] Srimathveeravalli G, Abdel-Atti D, Perez-Medina C, Takaki H, Solomon SB, Mulder WJM (2018). Reversible Electroporation-Mediated Liposomal Doxorubicin Delivery to Tumors Can Be Monitored With Zr-89-Labeled Reporter Nanoparticles. Mol Imaging.

[131] Phonesouk E, Lechevallier S, Ferrand A, Rols MP, Bezombes C, Verelst M (2019). Increasing Uptake of Silica Nanoparticles with Electroporation: From Cellular Characterization to Potential Applications. Materials.

[132] Petrella RA, Levit SL, Fesmire CC, Tang C, Sano MB (2022). Polymer Nanoparticles Enhance Irreversible Electroporation In Vitro. IEEE Trans Biomed Eng.

[133] Li N, Tang J, Yang J, Zhu B, Wang X, Luo Y (2021). Tumor perfusion enhancement by ultrasound stimulated microbubbles potentiates PD-L1 blockade of MC38 colon cancer in mice. Cancer Lett.

[134] Chen Y, Liang YB, Jiang P, Li F, Yu B, Yan F (2019). Lipid/PLGA Hybrid Microbubbles as a Versatile Platform for Noninvasive Image-Guided Targeted Drug Delivery. ACS Appl Mater Interfaces.

[135] Sun L, Zhang J, Xu M, Zhang L, Tang Q, Chen J (2022). Ultrasound Microbubbles Mediated Sonosensitizer and Antibody Co-delivery for Highly Efficient Synergistic Therapy on HER2-Positive Gastric Cancer. ACS Appl Mater Interfaces.

[136] Lan M, Zhu L, Wang Y, Shen D, Fang K, Liu Y (2020). Multifunctional nanobubbles carrying indocyanine green and paclitaxel for molecular imaging and the treatment of prostate cancer. J Nanobiotechnology.

[137] Liu Y, Zhou Y, Xu J, Luo H, Zhu Y, Zeng X (2021). Ultrasound molecular imaging-guided tumor gene therapy through dual-targeted cationic microbubbles. Biomater Sci.

